# Global, regional, and national burden of thyroid cancer in women of child-bearing age, 1990 to 2021 and predictions to 2035: An analysis of the global burden of disease study 2021

**DOI:** 10.3389/fendo.2025.1555841

**Published:** 2025-06-27

**Authors:** Tao Jiang, Li Bin, Huiting Liu, Cailiang Gao, Xue Liu

**Affiliations:** ^1^ Department of Nuclear Medicine, Hunan University of Medicine General Hospital, Huaihua, Hunan, China; ^2^ Department of Nuclear Medicine, Chongqing University Three Gorges Hospital, Wanzhou, Chongqing, China

**Keywords:** thyroid cancer, women of child-bearing age, DALYs, incidence, global burden of disease, socio-demographic index, trend prediction

## Abstract

**Background:**

Thyroid cancer has increased globally, particularly among young women, highlighting the need for research on its epidemiological characteristics and disease burden in women of child-bearing age. This study aimed to analyze the global and regional burden of thyroid cancer from 1990 to 2021, focusing on women of child-bearing age, and to predict trends up to 2035.

**Methods:**

This study analyzed the global and regional burden of thyroid cancer from 1990 to 2021, focusing on women of child-bearing age, using data from the Global Burden of Disease Study. Key indicators assessed included incidence, mortality, and disability-adjusted life years (DALYs) of thyroid cancer in different regions. Statistical analysis techniques were employed to compare the burden across regions and countries, examining the effects of age, sex, and socio-demographic index (SDI) on disease burden. The Bayesian Age-Period-Cohort model was used to predict the incidence, mortality, and DALYs of thyroid cancer from 2022 to 2035.

**Results:**

Globally, in 2021, there were 67,558 new cases of thyroid cancer among women of child-bearing age, with 3,260 deaths and 206,508 DALYs. Compared to 1990, new cases increased by 156.86%, deaths increased by 52.33%, and DALYs increased by 61.72%. The age-standardized incidence rate (ASIR), mortality rate (ASMR), and DALYs rate (ASDR) per 100,000 population were 3.37, 0.16, and 10.38, respectively. The Estimated Annual Percentage Change (EAPC) for ASIR was 1.47, indicating an increasing trend, whereas the EAPCs for ASMR and ASDR were -0.35 and -0.05, showing decreasing trends. The burden of thyroid cancer among patients with women of child-bearing age exhibited a significant age-related trend, peaking in the 45–49 age group. There were significant regional and national variations in thyroid cancer burden, which are closely related to the SDI. By 2035, a notable increase in the incidence, mortality, and DALYs associated with thyroid cancer among women of child-bearing age has been predicted globally.

**Conclusion:**

Over the past 30 years, thyroid cancer incidence among women has significantly increased globally, with slightly declining mortality and DALYs rates. Significant regional and national variations are closely linked to the SDI. As the population ages and incidence continues to rise, targeted prevention and treatment strategies, particularly in low SDI regions, are crucial to effectively reduce mortality and DALYs.

## 1 Introduction

In recent decades, the incidence of thyroid cancer has significantly increased globally, particularly among young women, making it a major public health issue that cannot be ignored (1, 2). According to data from the Global Burden of Disease (GBD) Study, thyroid cancer has become one of the most common endocrine tumors in women, with its continuously increasing incidence attracting widespread attention and concern from researchers and clinicians (3). Although the overall mortality rate of thyroid cancer is relatively low (4), the rising incidence and its potential impact on women of child-bearing age urgently require more in-depth and systematic research.

Currently, research on thyroid cancer primarily focuses on its pathogenesis, diagnostic methods, and treatment strategies (5, 6). However, systematic studies on the epidemiological characteristics and disease burden of thyroid cancer in women of child-bearing age remain relatively insufficiently comprehensive. In particular, comparative analyses of the incidence, mortality, and related burdens across different regions, countries, and globally are lacking, which limits the formulation and implementation of public health policies and affects the effective response to this disease (4, 7).

Furthermore, while some studies have explored sex differences and age-related factors in thyroid cancer, the specific impact on women of child-bearing age has not been comprehensively assessed (8, 9). In particular, women in countries with lower socioeconomic status may face higher disease burdens and lack necessary medical support and resources (10, 11). Therefore, filling this research gap will be of significant and far-reaching importance for improving women’s health and enhancing public health intervention measures.

This study aimed to analyze the global and regional burden of thyroid cancer from 1990 to 2021, with a particular focus on the disease burden among women of child-bearing age and to predict trends up to 2035. We will utilize data from the GDB study to assess key indicators, such as the incidence, mortality, and disability-adjusted life years (DALYs) of thyroid cancer in different regions. Through these data, we hope to reveal the epidemiological characteristics and changing trends of thyroid cancer in women of child-bearing age, thereby providing important references for future public health policies.

## 2 Methods

This study utilized data from GBD 2021 to analyze the global, regional, and national burden of thyroid cancer in women of child-bearing age. According to the World Health Organization, women of child-bearing age are defined as those aged 15–49 years.

### 2.1 Data sources and disease definitions

Thyroid cancer was classified under the International Classification of Diseases, 10 and 11 editions, with codes C73 and 2D10, respectively. In GBD 2021, thyroid cancer is categorized as a third-level cause of neoplasms. It encompasses various types of cancers, including papillary, follicular, medullary, and anaplastic thyroid cancers, although GBD 2021 does not differentiate between these subtypes.

GBD 2021 includes health data from 1990 to 2021, providing comprehensive and reliable data on the incidence, mortality, and DALYs of 459 diseases across 204 countries and regions. Data were accessed using the GBD 2021 Data Input Sources Tool and specifically extracted for women aged 15–49 years. All data are available online from the GBD results tool (https://vizhub.healthdata.org/gbd-results/).

### 2.2 Statistical analysis

#### 2.2.1 Age-standardized rates

To assess the age-standardized rates (ASR) of thyroid cancer in the women of child-bearing age, we employed a method that adjusts for age variations across populations. The ASR was calculated using the following formula:


$$\text{ASR}=\frac{\sum_{i=1}^{N}\alpha_{i}W_{i}}{\sum_{i=1}^{N}W_{i}}\times 100,000$$

Here, a_*i*_ denotes the age-specific rate for thyroid cancer among women of child-bearing age in the *i*th age group, where $W_{i}$ represents the corresponding population count (or weight) within the GBD standard population, and N is the total number of age groups considered. The disease burden of thyroid cancer in this population includes the number of incident cases, deaths, and DALYs, which reflects new diagnoses, fatalities, and comprehensive health impacts. To facilitate comparisons across populations, we also calculated the age-standardized incidence rate (ASIR), age-standardized mortality rate (ASMR), and age-standardized DALYs rate (ASDR), which were adjusted for population differences. The 95% uncertainty intervals (UIs) for these indicators are constructed by selecting the 2.5th and 97.5th percentiles from an ordered set of 1000 statistical draws using the ‘ageadjust.direct’ function from the ‘epitools’ package in R software, providing a reliable estimate of the variability in our calculations.

#### 2.2.2 Trend analysis

The Estimated Annual Percentage Change (EAPC) in the ASR was determined by fitting the natural logarithm of ASR to a linear regression model:


$$\text{ln}\left(\text{ASR}\right)=\text{α}+\text{β}x+\epsilon$$

where *x* represents the calendar year. The EAPC was then derived as:


$$\text{EAPC}=100\times \left({\text{ e}}^{\text{β}}-1\right)$$

A trend is significantly increasing if the EAPC is above zero and its 95% confidence intervals (95% CIs) do not include zero, decreasing if it is below zero and the interval does not include zero, and is considered constant if the 95% CIs include zero.

#### 2.2.3 Relationship with socio-demographic index

The socio-demographic index (SDI) is a composite measure that encapsulates a country’s developmental status and exhibits a strong correlation with health outcomes. It is calculated using the geometric mean of three standardized indicators: fertility rate among women under 25 years, average years of education for individuals aged 15 and above, and lag-adjusted income per capita. For the GBD 2021 study, the SDI values were scaled by multiplying them by 100 for reporting purposes. An SDI score of 0 signifies the lowest level of development relevant to health, whereas a score of 100 indicates the highest level. A recent GBD 2021 capstone paper detailed the methodology for constructing the SDI and categorized 204 countries into five developmental quintiles (low, low-middle, middle, high-middle, and high) based on their SDI estimates for the year 2021.

We examined the relationship between the burden of thyroid cancer among women of childbearing age and the SDI using local regression smoothing models (Loess) with the geom_smooth function from the ggplot2 package across 21 regions and 204 countries and territories. Spearman correlation analysis was conducted to determine the correlation coefficients (r) and p-values for the association between cancer burden and the SDI in 2021. Statistical significance was set at P < 0.05.

#### 2.2.4 Predictions using Bayesian age-period-cohort model

To understand the post-2021 trends in the GBD of thyroid cancer among women of child-bearing age, we employed the Bayesian Age-Period-Cohort (BAPC) model, which assumes a logarithmic linear Poisson framework, to predict incidence, mortality, and DALYs for thyroid cancer from 2022 to 2035 (12). Analyses were conducted using the BAPC package in R.

#### 2.2.5 Software and tools

All statistical analyses and graphical representations were performed using R software version 4.4.1 and JD_GBDR (V2.22, Jingding Medical Technology Co., Ltd.).

### 2.3 Ethics statement

The Institutional Review Board of the University of Washington reviewed and approved the waiver for informed consent (https://www.healthdata.org/research-analysis/gbd).

## 3 Results

### 3.1 Global level

**Figure 1:**
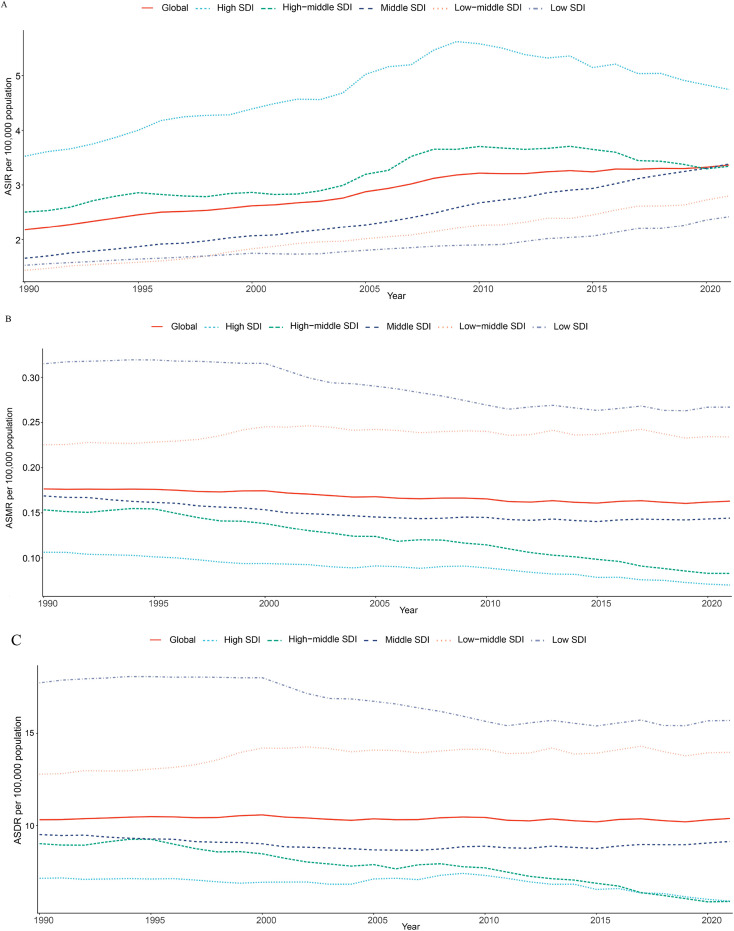
Time trends in ASIR **(A)**, ASMR **(B),** and ASDR **(C)** for thyroid cancer among women of childbearing age in the global and 5 SDI regions from 1990 to 2021. ASIR, age-standardized incidence rate; ASMR, age-standardized mortality rate; ASDR, age-standardized DALYs rate; DALYs, disability-adjusted life-years; SDI, Socio-demographic index.

**Figure 2:**
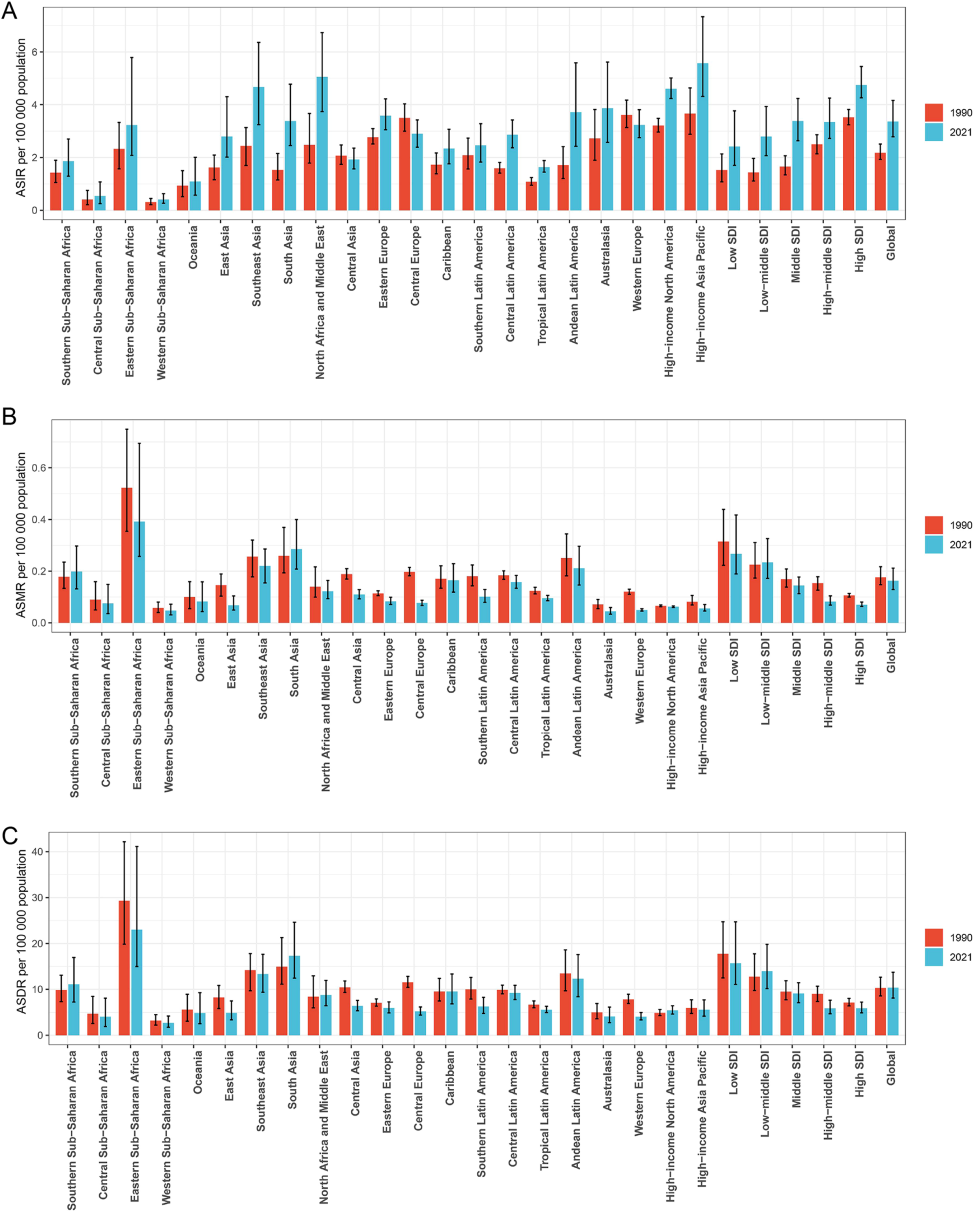
Histogram comparing the ASIR **(A)**, ASMR **(B)**, and ASDR **(C)** of thyroid cancer in women of childbearing age in 1990 and 2021 across the global, 21 regions, and 5 SDI regions. ASIR, age-standardized incidence rate; ASMR, age-standardized mortality rate; ASDR, age-standardized DALYs rate; DALYs, disability-adjusted life-years; SDI, Socio-demographic index.

Globally, in 2021, the number of new thyroid cancer cases among women of child-bearing age was 67,558, with 3,260 deaths and 206,508 DALYs (**Table 1**). Compared to 1990, the number of new cases, deaths, and DALYs increased by 156.86%, 52.33%, and 61.72%, respectively. The ASIR, ASMR, and ASDR per 100,000 population for thyroid cancer among women of child-bearing age in 2021 were 3.37, 0.16, and 10.38, respectively. From 1990 to 2021, the EAPC for ASIR was 1.47, indicating an increasing trend, while the EAPCs for ASMR and ASDR were -0.35 and -0.05, respectively, showing decreasing trends (**Table 1**; **Figures 1**, **2**).

**Table 1:** Incidence, death, and DALYs of thyroid cancer in women of childbearing age (15-49 Years) globally, in 5 SDI regions, and 21 GBD regions in 1990 and 2021, with estimated annual percentage changes from 1990 to 2021.

Characteristics	1990						2021						EAPC (1990–2021)		
	Cases			Rates			Cases			Rates					
	Incidence(95% UI)	Deaths (95% UI)	DALYs (95% UI)	ASIR per 100,000 (95% UI)	ASMR per 100,000 (95% UI)	ASDR per 100,000 (95% UI)	Incidence (95% UI)	Deaths (95% UI)	DALYs (95% UI)	ASIR per 100,000 (95% UI)	ASMR per 100,000 (95% UI)	ASDR per 100,000 (95% UI)	ASIR (95% CI)	ASMR (95% CI)	ASDR (95% CI)
**Global**	26,302 (23,183-30,421)	2140 (1778-2648)	127,692 (105,583-157,611)	2.18 (1.93-2.51)	0.18 (0.15-0.22)	10.31 (8.58-12.66)	67,558 (55,974-83,415)	3,260 (2,578-4,215)	206,508 (161,917-272,323)	3.37 (2.78-4.16)	0.16 (0.13-0.21)	10.38 (8.13-13.74)	1.47 (1.34 to 1.59)	-0.35 (-0.38 to -0.31)	-0.05 (-0.08 to -0.02)
SDI region															
High SDI	8,327 (7,657-9,027)	250 (235-267)	16,763 (15,073-18,850)	3.53 (3.24-3.82)	0.11 (0.10-0.11)	7.13 (6.41-8.02)	13,395 (12,062-15,305)	200 (180-229)	16,546 (13,790-20,260)	4.75 (4.26-5.44)	0.07 (0.06-0.08)	5.89 (4.90-7.24)	1.22 (0.90 to 1.54)	-1.23 (-1.33 to -1.12)	-0.42 (-0.58 to -0.25)
High-middle SDI	6,462 (5,517-7,414)	392 (321-457)	23,383 (19,074-27,804)	2.50 (2.14-2.87)	0.15 (0.13-0.18)	9.02 (7.38-10.69)	12,094 (9,893-15,317)	304 (252-381)	21,086 (16,910-27,230)	3.34 (2.72-4.25)	0.08 (0.07-0.10)	5.88 (4.70-7.65)	1.20 (0.93 to 1.46)	-2.09 (-2.23 to -1.95)	-1.40 (-1.55 to -1.26)
Middle SDI	6,410 (5,165-7,995)	638 (520-792)	37,089 (30,013-46,485)	1.66 (1.34-2.06)	0.17 (0.14-0.21)	9.51 (7.72-11.87)	22,327 (17,413-27,878)	959 (751-1,172)	59,997 (46,706-75,041)	3.38 (2.63-4.23)	0.14 (0.11-0.18)	9.14 (7.10-11.47)	2.36 (2.30 to 2.41)	-0.57 (-0.67 to -0.48)	-0.18 (-0.27 to -0.09)
Low-middle SDI	3,538 (2,712-4,875)	548 (418-761)	32,136 (24,376-44,917)	1.44 (1.11-1.97)	0.23 (0.17-0.31)	12.78 (9.78-17.76)	13,681 (10,073-19,367)	1,140 (832-1,599)	68,877 (49,898-98,395)	2.80 (2.07-3.93)	0.23 (0.17-0.33)	13.97 (10.17-19.82)	2.17 (2.11 to 2.23)	0.12 (0.03 to 0.22)	0.27 (0.17 to 0.37)
Low SDI	1,532 (1,079-2,144)	310 (218-432)	18,192 (12,728-25,298)	1.53 (1.08-2.14)	0.31 (0.22-0.44)	17.76 (12.50-24.75)	6,009 (4,229-9,441)	655 (462-1,030)	39,857 (27,990-63,165)	2.42 (1.71-3.77)	0.27 (0.19-0.42)	15.70 (11.07-24.73)	1.31 (1.21 to 1.41)	-0.79 (-0.88 to -0.69)	-0.64 (-0.73 to -0.55)
GBD region															
Andean Latin America	135 (94-192)	19 (14-27)	1,074 (766-1,490)	1.71 (1.20-2.41)	0.25 (0.18-0.34)	13.49 (9.69-18.61)	644 (420-968)	36 (25-51)	2,134 (1,456-3,051)	3.72 (2.43-5.58)	0.21 (0.15-0.30)	12.33 (8.42-17.59)	2.53 (2.29 to 2.76)	-0.66 (-0.79 to -0.53)	-0.39 (-0.52 to -0.25)
Australasia	149 (104-210)	4 (3-5)	274 (196-377)	2.72 (1.90-3.82)	0.07 (0.06-0.09)	5.01 (3.59-6.90)	317 (212-460)	4 (3-5)	337 (226-501)	3.87 (2.58-5.62)	0.04 (0.03-0.06)	4.12 (2.74-6.14)	1.79 (0.76 to 2.84)	-0.95 (-1.75 to -0.15)	-0.00 (-0.88 to 0.88)
Caribbean	142 (113-178)	14 (11-18)	790 (615-1,033)	1.73 (1.38-2.17)	0.17 (0.13-0.22)	9.54 (7.48-12.39)	288 (217-377)	20 (15-28)	1,172 (842-1,637)	2.34 (1.76-3.07)	0.17 (0.12-0.23)	9.55 (6.84-13.36)	1.09 (0.86 to 1.32)	0.03 (-0.24 to 0.31)	0.15 (-0.11 to 0.42)
Central Asia	286 (240-341)	25 (22-28)	1,445 (1,285-1,632)	2.07 (1.74-2.48)	0.19 (0.17-0.21)	10.48 (9.33-11.83)	486 (395-593)	27 (23-32)	1,610 (1,343-1,910)	1.93 (1.57-2.35)	0.11 (0.09-0.13)	6.42 (5.36-7.61)	-0.23 (-1.05 to 0.60)	-2.08 (-2.74 to -1.41)	-1.86 (-2.53 to -1.18)
Central Europe	1,135 (971-1,306)	63 (59-69)	3,715 (3,357-4,129)	3.50 (3.00-4.03)	0.20 (0.18-0.21)	11.55 (10.44-12.84)	943 (778-1,112)	26 (23-29)	1,705 (1,434-2,010)	2.90 (2.39-3.43)	0.08 (0.07-0.09)	5.24 (4.39-6.19)	-0.86 (-1.05 to -0.67)	-3.33 (-3.54 to -3.12)	-2.81 (-3.02 to -2.61)
Central Latin America	541 (478-618)	60 (55-66)	3,366 (3,060-3,730)	1.59 (1.41-1.81)	0.18 (0.17-0.20)	9.92 (9.05-10.94)	1,993 (1,650-2,382)	110 (94-128)	6,427 (5,430-7,589)	2.86 (2.37-3.42)	0.16 (0.13-0.18)	9.22 (7.79-10.89)	1.90 (1.69 to 2.11)	-0.43 (-0.77 to -0.08)	-0.18 (-0.51 to 0.16)
High-income Asia Pacific	1,797 (1,414-2,268)	41 (34-52)	2,908 (2,348-3,751)	3.67 (2.88-4.64)	0.08 (0.07-0.11)	5.94 (4.77-7.72)	2,689 (2,086-3,508)	28 (23-36)	2,702 (2,024-3,700)	5.57 (4.31-7.34)	0.06 (0.05-0.07)	5.59 (4.15-7.70)	1.75 (1.11 to 2.39)	-0.96 (-1.42 to -0.50)	0.16 (-0.36 to 0.68)
Central Sub-Saharan Africa	41 (21-76)	9 (5-16)	475 (256-862)	0.41 (0.21-0.75)	0.09 (0.05-0.16)	4.72 (2.58-8.47)	148 (69-296)	20 (9-40)	1,113 (521-2,242)	0.54 (0.26-1.08)	0.08 (0.04-0.15)	4.06 (1.91-8.09)	0.93 (0.64 to 1.22)	-0.56 (-0.70 to -0.42)	-0.48 (-0.63 to -0.33)
East Asia	4,777 (3,411-6,166)	418 (296-547)	24,353 (17,201-32,120)	1.63 (1.16-2.09)	0.15 (0.10-0.19)	8.26 (5.84-10.85)	10,949 (7,890-16,774)	272 (195-412)	19,022 (13,145-28,903)	2.80 (2.01-4.31)	0.07 (0.05-0.10)	4.90 (3.38-7.49)	1.79 (1.66 to 1.93)	-2.79 (-2.93 to -2.64)	-1.99 (-2.13 to -1.84)
Eastern Europe	1,571 (1,427-1,757)	63 (59-69)	3,955 (3,549-4,451)	2.77 (2.51-3.10)	0.11 (0.11-0.12)	7.06 (6.33-7.93)	2,157 (1,841-2,536)	51 (44-61)	3,568 (2,937-4,375)	3.58 (3.05-4.22)	0.08 (0.07-0.10)	5.94 (4.89-7.30)	1.77 (0.90 to 2.65)	-0.49 (-1.29 to 0.31)	0.08 (-0.75 to 0.91)
High-income North America	2,483 (2,293-2,696)	50 (48-53)	3,762 (3,303-4,332)	3.21 (2.97-3.49)	0.07 (0.06-0.07)	4.90 (4.30-5.63)	4,256 (3,912-4,630)	58 (55-61)	4,996 (4,259-5,943)	4.60 (4.23-5.01)	0.06 (0.06-0.07)	5.43 (4.63-6.45)	1.33 (1.15 to 1.52)	-0.03 (-0.16 to 0.10)	0.49 (0.36 to 0.62)
Eastern Sub-Saharan Africa	880 (591-1,259)	192 (129-277)	11,351 (7,630-16,343)	2.33 (1.57-3.33)	0.52 (0.35-0.75)	29.33 (19.84-42.17)	3,141 (2,009-5,644)	373 (244-664)	22,843 (14,804-40,928)	3.23 (2.08-5.78)	0.39 (0.26-0.69)	23.01 (14.98-41.12)	0.74 (0.53 to 0.95)	-1.30 (-1.48 to -1.12)	-1.16 (-1.34 to -0.98)
North Africa and Middle East	1,633 (1,172-2,415)	90 (64-139)	5,585 (3,971-8,623)	2.48 (1.79-3.67)	0.14 (0.10-0.22)	8.41 (5.99-12.95)	8,156 (6,023-1,0847)	196 (149-263)	14,110 (10,369-19,187)	5.06 (3.74-6.73)	0.12 (0.09-0.16)	8.80 (6.47-11.97)	2.72 (2.56 to 2.88)	-0.13 (-0.25 to -0.01)	0.47 (0.34 to 0.61)
Oceania	12 (7-20)	1 (1-2)	74 (40-118)	0.93 (0.52-1.50)	0.10 (0.06-0.16)	5.60 (3.06-8.94)	36 (19-66)	3 (1-5)	160 (83-305)	1.09 (0.57-2.01)	0.08 (0.04-0.16)	4.87 (2.52-9.29)	0.32 (0.17 to 0.46)	-0.59 (-0.63 to -0.54)	-0.47 (-0.52 to -0.42)
South Asia	3,673 (2,742-5,174)	611 (454-872)	36,363 (26,976-51,820)	1.53 (1.15-2.15)	0.26 (0.19-0.37)	14.96 (11.15-21.27)	16,419 (11,891-23,303)	1,380 (1,003-1,941)	84,402 (60,512-120,598)	3.38 (2.45-4.78)	0.29 (0.21-0.40)	17.31 (12.43-24.61)	2.67 (2.59 to 2.74)	0.32 (0.21 to 0.42)	0.47 (0.35 to 0.58)
Southeast Asia	2,536 (1,737-3,274)	255 (174-320)	14,593 (9,838-18,431)	2.45 (1.70-3.13)	0.26 (0.18-0.32)	14.20 (9.71-17.81)	8,935 (6,220-1,2134)	426 (299-551)	25,611 (17,970-33,778)	4.67 (3.25-6.36)	0.22 (0.15-0.29)	13.35 (9.37-17.64)	1.92 (1.78 to 2.06)	-0.57 (-0.64 to -0.51)	-0.31 (-0.38 to -0.24)
Southern Latin America	249 (187-327)	21 (17-27)	1,196 (943-1,508)	2.09 (1.57-2.73)	0.18 (0.14-0.22)	10.01 (7.91-12.61)	457 (339-608)	19 (15-24)	1,163 (886-1,527)	2.46 (1.82-3.28)	0.10 (0.08-0.13)	6.28 (4.78-8.26)	0.72 (0.45 to 0.98)	-1.67 (-2.01 to -1.32)	-1.30 (-1.64 to -0.97)
Southern Sub-Saharan Africa	158 (116-212)	19 (14-25)	1,100 (818-1,471)	1.42 (1.05-1.90)	0.18 (0.13-0.23)	9.85 (7.35-13.09)	392 (271-571)	41 (27-62)	2,336 (1,514-3,582)	1.86 (1.29-2.70)	0.20 (0.13-0.30)	11.12 (7.25-16.96)	1.52 (0.89 to 2.16)	1.22 (0.62 to 1.83)	1.24 (0.59 to 1.89)
Tropical Latin America	373 (327-428)	42 (38-46)	2,300 (2,063-2,586)	1.08 (0.95-1.24)	0.12 (0.11-0.14)	6.68 (6.01-7.50)	1,078 (951-1,238)	63 (57-70)	3,652 (3,245-4,137)	1.64 (1.44-1.88)	0.10 (0.09-0.11)	5.57 (4.95-6.31)	1.02 (0.71 to 1.32)	-1.10 (-1.29 to -0.90)	-0.86 (-1.08 to -0.65)
Western Europe	3,614 (3,136-4,174)	121 (111-131)	7,812 (6,898-8,929)	3.61 (3.13-4.17)	0.12 (0.11-0.13)	7.82 (6.91-8.95)	3,648 (3,115-4,284)	57 (53-62)	4,569 (3,787-5,557)	3.23 (2.75-3.81)	0.05 (0.05-0.05)	4.06 (3.35-4.95)	-0.10 (-0.56 to 0.36)	-2.67 (-2.87 to -2.46)	-1.88 (-2.15 to -1.61)
Western Sub-Saharan Africa	116 (78-168)	21 (14-29)	1,201 (812-1,717)	0.32 (0.22-0.45)	0.06 (0.04-0.08)	3.22 (2.21-4.52)	426 (278-664)	48 (32-75)	2,877 (1,870-4,504)	0.41 (0.27-0.63)	0.05 (0.03-0.07)	2.71 (1.77-4.20)	0.80 (0.68 to 0.91)	-0.74 (-0.83 to -0.64)	-0.59 (-0.68 to -0.49)

GBD, Global Burden of Disease; ASIR, age-standardized incidence rate; ASMR, age-standardized mortality rate; ASDR, age-standardized DALY rate; DALYs, disability-adjusted life-years; EAPC, estimated annual percentage change; SDI, Socio-demographic index; UI, uncertainty interval; CI, confidence interval.

**Figure 3:**
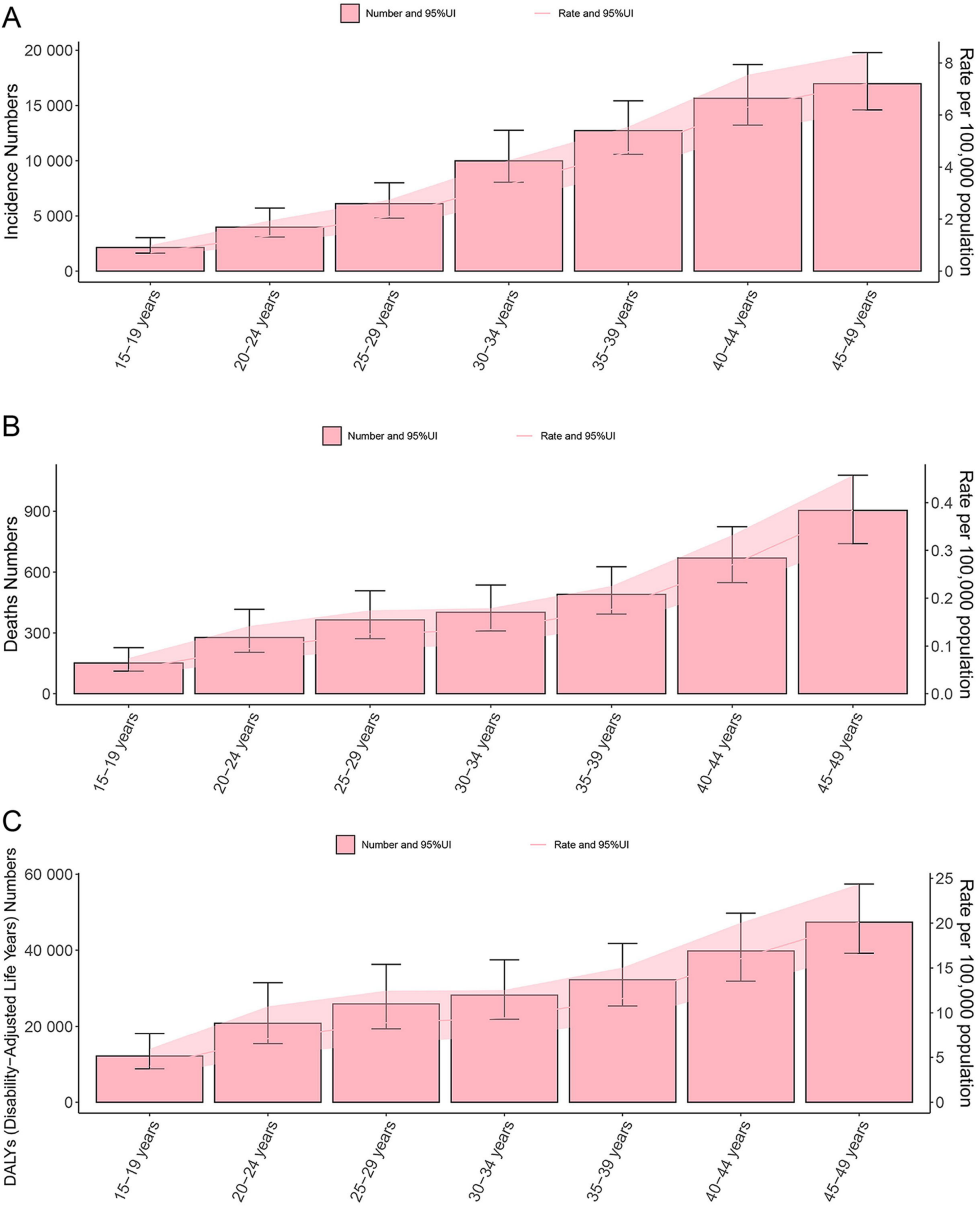
Double plot of age-specific burden of thyroid cancer in women of childbearing age, incidence and age-standardized incidence rate **(A)**, deaths and age-standardized mortality rate **(B)**, DALYs and age-standardized DALYs rate **(C)**, in 2021. DALYs, disability-adjusted life years; UI, uncertainty interval.

**Figure 4:**
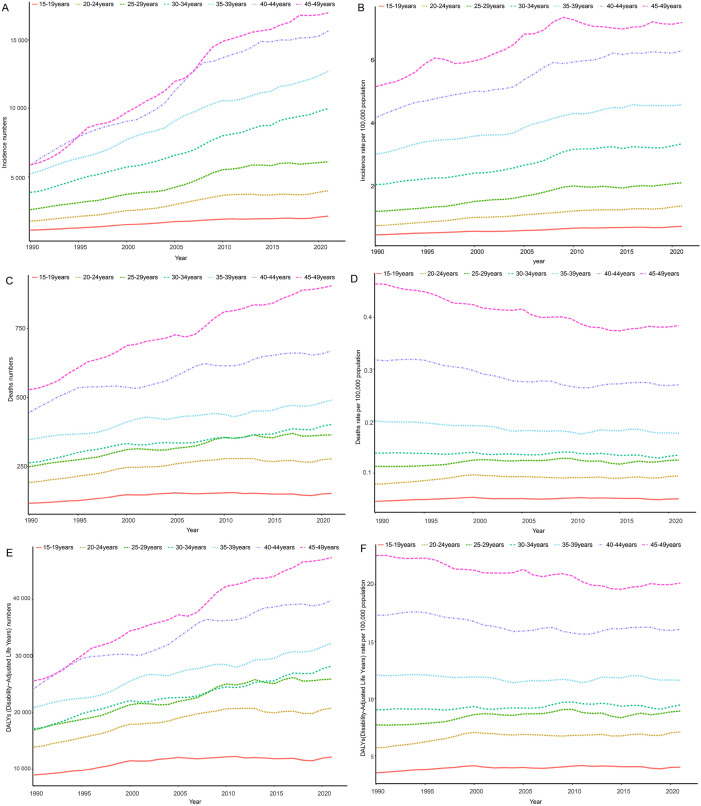
Time trends in incidence **(A, B)**, death **(C, D)**, and DALYs **(E, F)** for age-specific thyroid cancer among women of childbearing age in the global from 1990 to 2021. DALYs, disability-adjusted life-years.

Globally, the disease burden of thyroid cancer among women of child-bearing age exhibits a significant age-related trend in 2021. When analyzed in 5-year age groups, the number and rate of new cases, deaths, and DALYs increased with age, peaking in the 45–49 age group (**Figure 3**). Specifically, the incidence rate in the 45–49 age group accounted for 30.8% of all age groups, mortality accounted for 34.2%, and DALYs accounted for 28.9%, highlighting the high concentration of risk, mortality, and health impact of thyroid cancer in this age group (**Supplementary Figure 1**). The temporal trend in thyroid cancer burden among women of child-bearing age at specific ages from 1990 to 2021 is shown in **Figure 4**.

### 3.2 5 SDI regional level

Among the five SDI regions, the highest number of new thyroid cancer cases among women of child-bearing age in 2021 was observed in the Middle SDI region (22,327) and the lowest was in the Low SDI region (6,009) (**Table 1**). Compared to 1990, all regions showed an increase, with the Low SDI region experiencing the fastest growth at 292.127% and the High SDI region experiencing the slowest growth at 60.868%. The region with the highest ASIR was the High SDI region (4.75/100,000) and the lowest was the Low SDI region (2.42/100,000). From 1990 to 2021, the ASIR increased in all SDI regions, with the Middle SDI region showing the most significant increase, with an EAPC of 2.36 (**Table 1**; **Figures 1**, **2**).

The regions with the highest number of deaths and DALYs were both the Low-middle SDI region, with 1,140 deaths and 68,877 DALYs, respectively, and the lowest were in the High SDI region (200 deaths and 16,546 DALYs) (**Table 1**). Compared to 1990, the Low-middle SDI, Low SDI, and Middle SDI regions showed increasing trends, whereas the High SDI and High-middle SDI regions showed negative growth. The Low SDI region had the highest ASMR and ASDR per 100,000 population, the High SDI region had the lowest ASMR (0.07/100,000), and the High-middle SDI region had the lowest ASDR (5.88/100,000). From 1990 to 2021, the ASMR and ASDR per 100,000 population increased in the Low-middle SDI region, whereas they decreased in the other SDI regions, with the High-middle SDI region showing the largest decrease, with EAPCs of -2.09 and -1.40, respectively (**Table 1**; **Figures 1**, **2**).

### 3.3 21 GBD regional level

Among the 21 GBD regions, in 2021, the regions with higher numbers of new thyroid cancer cases among women of child-bearing age were East Asia (10,949), Southeast Asia (8,935), North Africa, and the Middle East (8,156), while the lower regions were the Caribbean (288), Central Sub-Saharan Africa (148), and Oceania (36). The regions with higher ASIR per 100,000 population were High-income Asia Pacific (5.57), North Africa and the Middle East (5.06), and Southeast Asia (4.67), while Western Sub-Saharan Africa (0.41), Central Sub-Saharan Africa (0.54), and Oceania (1.09) had the lowest rates (**Table 1**; **Figure 2**).

The regions with higher numbers of deaths were South Asia (1,380), Southeast Asia (426), and Eastern Sub-Saharan Africa (373), while the regions with lower numbers of deaths were Southern Latin America (19), Australasia (4), and Oceania (3). The regions with higher age-standardized mortality rates per 100,000 population were Eastern Sub-Saharan Africa (0.392), South Asia (0.286), and Southeast Asia (0.221), while Australasia (0.045), Western Sub-Saharan Africa (0.047), and Western Europe (0.050) had the lowest rates (**Table 1**; **Figure 2**).

The regions with higher DALYs were South Asia (84,402), Southeast Asia (25,611), and Eastern Sub-Saharan Africa (22,843), while the regions with lower DALYs were Central Sub-Saharan Africa (1,113), Australasia (337), and Oceania (160). The regions with higher age-standardized DALYs rates per 100,000 population were Eastern Sub-Saharan Africa (23.01), South Asia (17.31), and Southeast Asia (13.35), while Central Sub-Saharan Africa (4.06), Western Europe (4.06), and Western Sub-Saharan Africa (2.71) had the lowest rates (**Table 1**; **Figure 2**).

From 1990 to 2021, the ASIR for thyroid cancer among women of child-bearing age showed a decreasing trend in Central Europe, with an EAPC of -0.863, while it remained stable in Western Europe and Central Asia. An increasing trend was observed in all the other regions. Meanwhile, the ASMR showed an increasing trend in Southern Sub-Saharan Africa and South Asia, with EAPCs of 1.223 and 0.316, respectively, while it remained stable in the Caribbean and High-income North America. A decreasing trend was observed in all the other regions. In terms of ASDR, Southern Sub-Saharan Africa showed the most significant increase, with an EAPC of 1.239, while Central Europe showed the most significant decrease, with an EAPC of -2.813. High-income Asia Pacific, the Caribbean, Eastern Europe, Australasia, and Central Latin America showed stable trends (**Table 1**; **Figure 2**).

### 3.4 Country level

**Figure 5:**
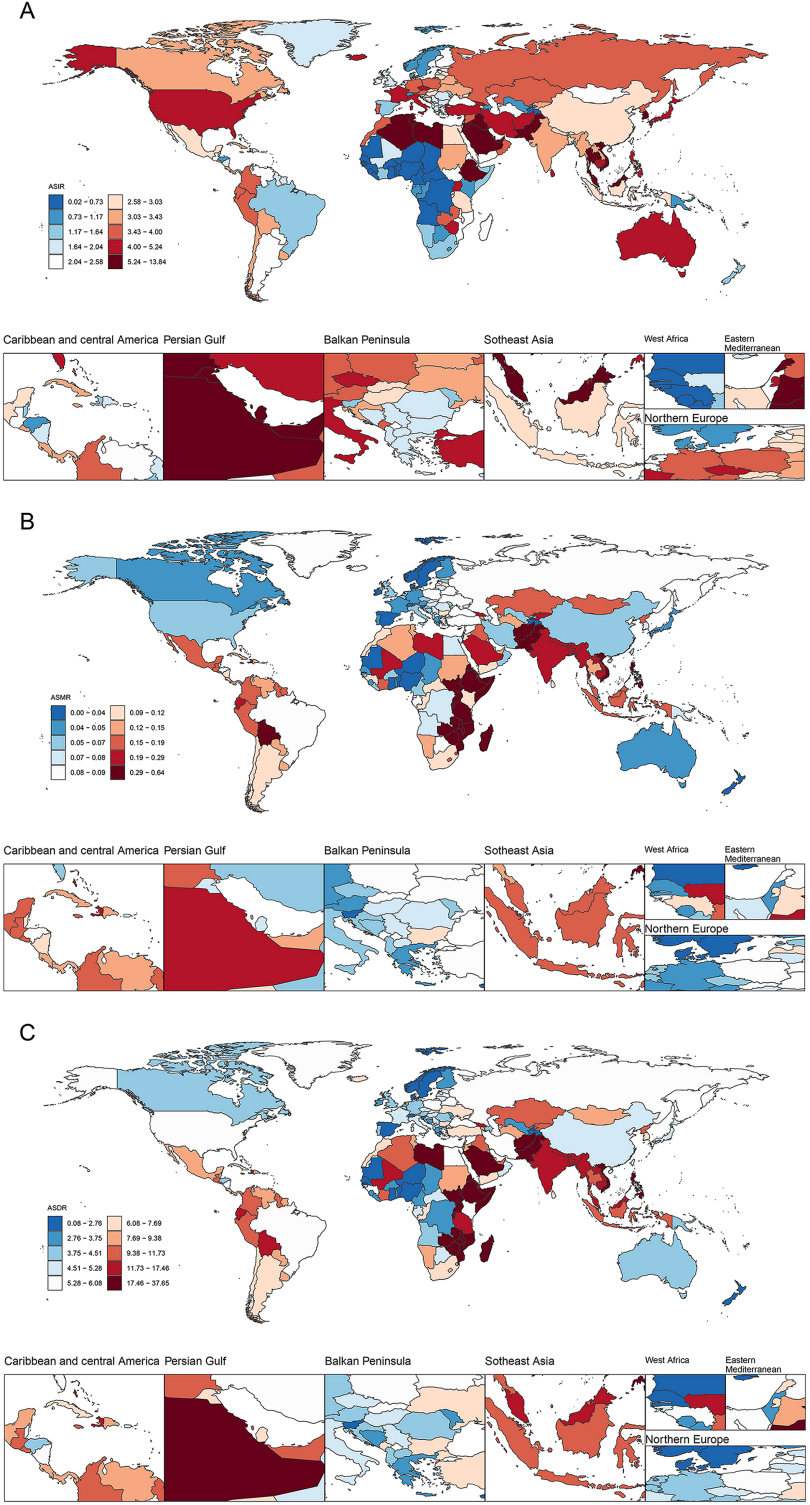
ASIR **(A)**, ASMR **(B)**, and ASDR **(C)** of thyroid cancer in women of childbearing age across 204 countries worldwide in 2021. ASIR, age-standardized incidence rate; ASMR, age-standardized mortality rate; ASDR, age-standardized DALYs rate; DALYs, disability-adjusted life-years.

At the country level in 2021, the country with the highest number of new thyroid cancer cases among women of child-bearing age was India (11,727), followed by China (10,017), and the United States (3,938). The country with the highest number of deaths was India (928), followed by Pakistan (334) and China (251). Similarly, India also had the highest number of DALYs (56,440), followed by Pakistan (20,614) and China (17,517) (**Figure 5**; **Supplementary Table 1**).

Similarly, Saudi Arabia had the highest ASIR of thyroid cancer per 100,000 population among women of child-bearing age, with a rate of 13.07, followed closely by Libya (11.73) and Vietnam (9.83). From 1990 to 2021, Saudi Arabia (EAPC=4.92), Iran (4.51), and Vietnam (4.49) have exhibited notable upward trends and swiftest growth rates in this regard. Regarding the ASMR per 100,000 population, Ethiopia led to a rate of 0.63, followed by Zimbabwe (0.62), and Pakistan (0.57). From 1990 to 2021, Zimbabwe (EAPC = 3.87), Lesotho (3.85), and Guam (2.92) demonstrated the fastest increase in ASMR. As for the ASDR per 100,000 population, Ethiopia topped the list at a rate of 37.27, followed by Pakistan (34.60) and Zimbabwe (34.41). Between 1990 and 2021, Zimbabwe (EAPC = 3.89), Lesotho (3.86), and Guam (3.17) showed the most rapid increases in ASDR (**Supplementary Table 1**; **Supplementary Figures 2, 3**).

### 3.5 The association between thyroid cancer burden and SDI

**Figure 6:**
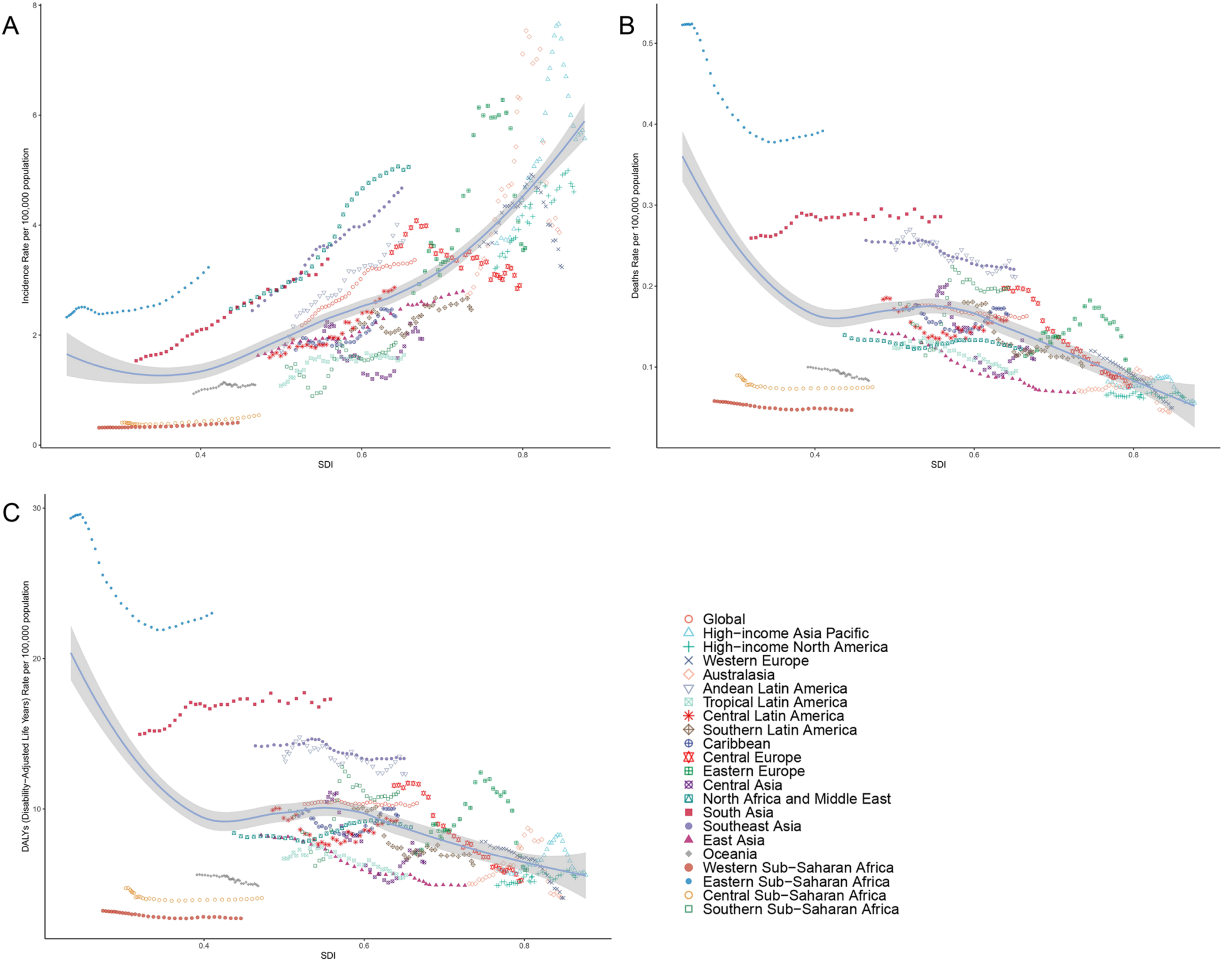
Correlation between thyroid cancer in women of childbearing age in ASIR **(A)**, ASMR **(B)** and ASDR **(C)** and SDI globally in 21 GBD regions from 1990 to 2021. ASMR, age-standardized mortality rate; ASDR, age-standardized DALY rate; GBD, global burden of disease study; DALYs, disability-adjusted life-years.

From 1990 to 2021, across the 21 GBD regions, the ASIR of thyroid cancer displayed a positive correlation with the SDI, indicating a gradual upward trend (r=0.7434, p<0.001). In stark contrast, both ASMR and ASDR exhibited negative correlations with SDI, suggesting a slow downward trend (r=-0.3652, -0.1877, both p<0.001). As the SDI increased globally, the ASMR and ASDR remained relatively stable, whereas the ASIR notably increased, surpassing the expected levels over the past three decades (**Figure 6**).

**Figure 7:**
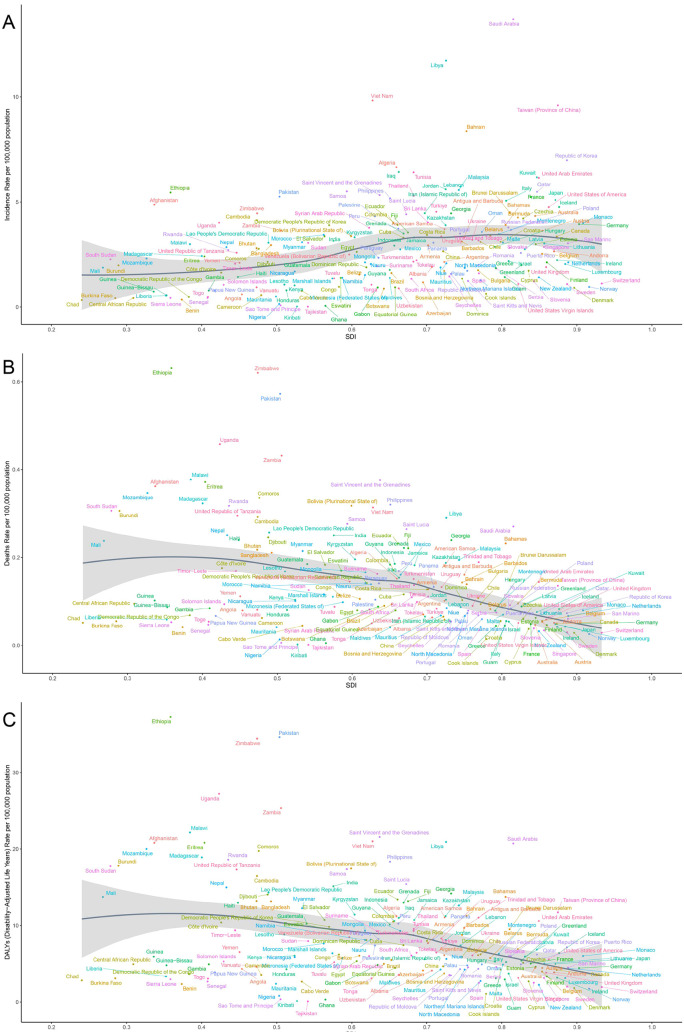
Correlation between thyroid cancer in women of childbearing age in ASIR **(A)**, ASMR **(B)** and ASDR **(C)** and SDI across 204 countries worldwide from 1990 to 2021. ASMR, age-standardized mortality rate; ASDR, age-standardized DALY rate; GBD, global burden of disease study; DALYs, disability-adjusted life-years.

Similarly, in 2021, across 204 countries and territories, the ASIR of thyroid cancer was positively correlated with the SDI, pointing to a gradual increase in incidence among higher SDI populations. Conversely, both ASMR and ASDR were negatively correlated with SDI (r=-0.430, -0.3035, both p<0.001) (**Figure 7**).

### 3.6 Bayesian prediction model

**Figure 8:**
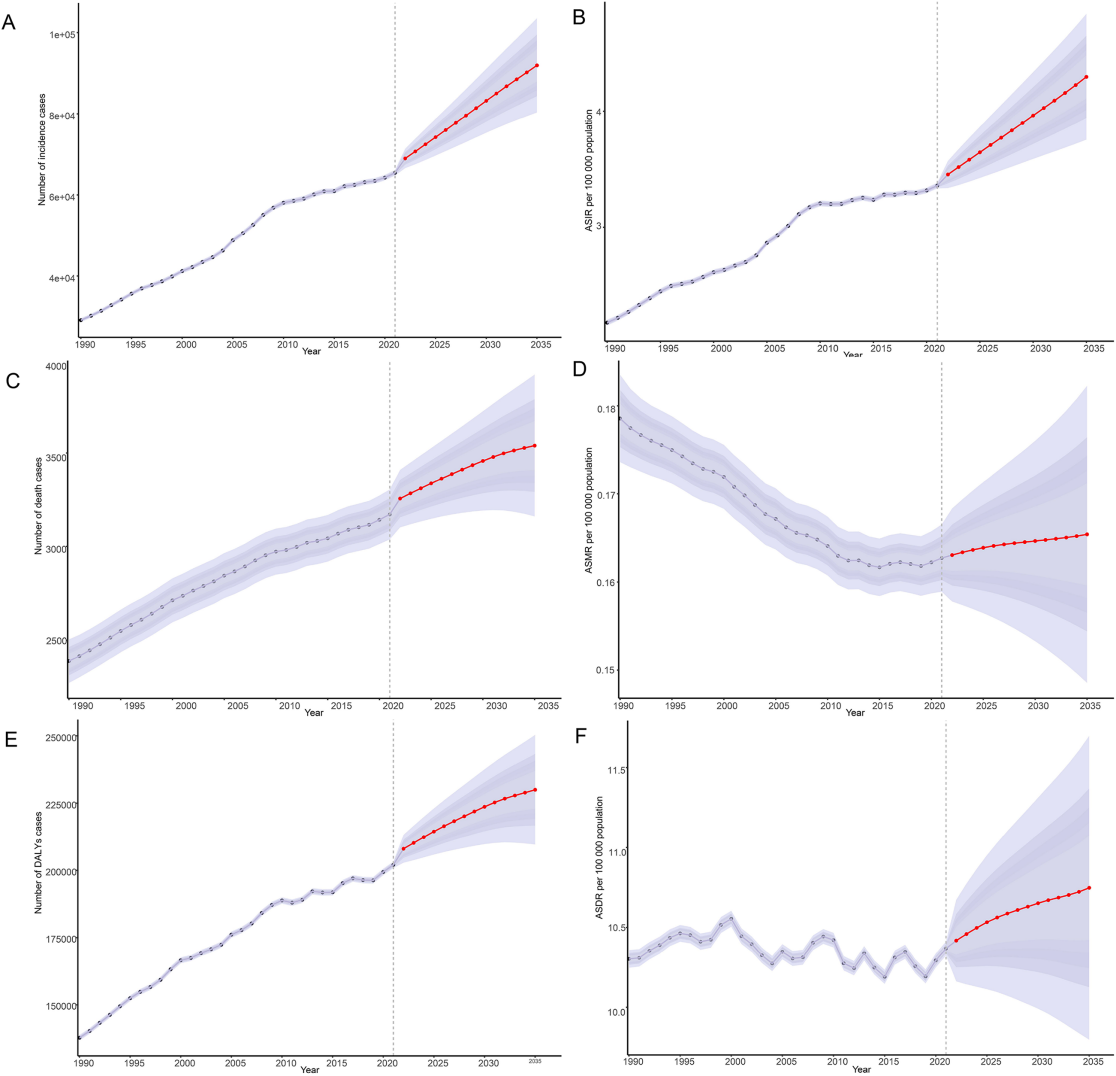
Predictions of the burden of thyroid cancer in women of childbearing age for 2035: Incidence and age-standardized incidence rate **(A, B)**, deaths and age-standardized mortality rate **(C, D)**, DALYs and age-standardized DALYs rate **(E, F)**, based on global data; DALYs, disability-adjusted life-years.

By 2035, there has been a notable increase in the incidence, mortality, and DALYs associated with thyroid cancer among women of child-bearing age globally (**Figure 8**). Specifically, the ASIR of thyroid cancer has been rising annually since 2021, increasing from 3.36/100,000 to 4.30/100,000 by 2035, with an EAPC of 1.68. Among these, the increase in incidence is most notable among women aged 45-49, rising from 7.20/100,000 in 2021 to 9.15/100,000 by 2035, with an EAPC of 1.77 (**Figure 8**; **Supplementary Figure 4**). However, despite the significant increase in incidence, there has been no notable change in ASMR and ASDR for thyroid cancer, suggesting that although the number of cases has increased, improvements in treatment efficacy and survival rates may have reduced mortality and the burden of DALYs (**Supplementary Figures 5, 6**).

## 4 Discussion

### 4.1 Evolution of the global burden of thyroid cancer

The results of this study clearly demonstrate that since 1990, there has been a significant global increase in the number of new cases, deaths, and DALYs due to thyroid cancer among women of child-bearing age. Specifically, the number of new thyroid cancer cases has increased by 156.86%, a trend that may be closely related to global population growth, aging, increased environmental exposure, changes in lifestyle, and continuous advancements in diagnostic techniques (13, 14). Additionally, the development of imaging technologies such as ultrasonography has greatly improved the detection rate of thyroid cancer, becoming one of the significant factors contributing to the surge in case numbers (15–17). While overdiagnosis and overtreatment may contribute to the increasing trend of thyroid cancer, it is noteworthy that these factors may not fully explain the upward trend (7). However, despite a significant rise in age-standardized incidence rates, there has been a decline in age-standardized mortality and DALYs rates, which likely reflects advancements in early detection systems and therapeutic efficacy (16, 18). Specifically, in upper-middle and high-income countries, improved diagnostic protocols for primary tumors and lymph node metastases have enabled timely interventions, contributing to survival rates comparable to the general population (19). This progress underscores the importance of prioritizing investments in early detection infrastructure, particularly for identifying metastatic spread.

The study found that the burden of thyroid cancer among women of child-bearing age varies significantly across different age groups, with a gradual increase as age increases. The incidence rate, mortality rate, and proportion of DALYs were highest in the 45–49 age group. This phenomenon may be related to hormonal fluctuations during the perimenopausal period, age-related changes in the thyroid structure, and the high detection rate resulting from frequent health examinations (16, 20–25). Therefore, implementing screening and prevention strategies for thyroid cancer among women in this age group is particularly important.

### 4.2 Association between regional disparities and SDI

There are significant regional disparities in the global burden of thyroid cancer among women of child-bearing age, which is consistent with the findings of Gong et al. (26) and Cheng et al. (27). Our study revealed that East Asia, Southeast Asia, North Africa, and the Middle East have the highest incidence rates, while Western Sub-Saharan Africa and Oceania have relatively lower incidence rates. Notably, neighboring regions with similar SDI levels often cluster in trends of thyroid cancer outcomes, underscoring the pivotal role of SDI in capturing contextual disparities in healthcare access and diagnostic practices (28). The regions with the highest mortality rates are concentrated in East Sub-Saharan Africa, South Asia, and Southeast Asia, which face challenges in their health care systems. Importantly, even modest economic improvements in low-resource settings may lead to a marked increase in thyroid cancer detection rates, reflecting enhanced diagnostic capacity rather than true biological risk escalation (28). A study (29) on the risk of thyroid cancer among Ontario and Canadian residents found that immigrants from Southeast Asia and East Asia have significantly higher incidence rates of thyroid cancer than immigrants from other regions and non-immigrants. Specifically, the risk ratio for thyroid cancer among Southeast Asian immigrants is as high as 2.66 times, and 1.87 times for East Asian immigrants compared to non-immigrants. These differences are attributed to various factors, including genetics, environment, nutrition, and regional variations in diagnostic protocols for thyroid nodule assessment (28, 29). Studies have suggested that differences in isoflavone and antioxidant vitamin intake significantly contribute to the relative attributable risk of thyroid cancer among Southeast Asian women (30).

Furthermore, there are notable disparities in the burden of thyroid cancer across the different SDI regions. This study finds that the Middle SDI regions have the highest number of new cases, while the Low SDI regions experience the most rapid growth. Notably, both Low-middle SDI and Low SDI regions showed an increasing trend in deaths and DALYs. In Low SDI regions, limited medical resources and weaker screening and diagnostic capabilities may lead to more undetected or unreported cases, underestimating the actual incidence rates (31). Additionally, high environmental exposure risks, such as air pollution and poor drinking water quality, may increase the incidence of thyroid cancer in these regions (32). The burden of thyroid cancer among aging populations has also significantly increased, indicating that these groups may lack effective health interventions (31). Furthermore, low healthcare investment and unequal resource distribution exacerbate inadequate management of thyroid cancer and other diseases, leading to increased mortality rates (31). International collaborative research emphasizes the importance of identifying relevant factors and effectively managing the global burden of thyroid cancer (31).

It is critical to acknowledge that while thyroid cancer incidence is rising disproportionately in women, these tumors often exhibit less aggressive biological behavior compared to those in men—a disparity potentially linked to hormonal influences on tumorigenesis and immune microenvironment regulation (33). Further exploring this gender difference, recent studies highlight its correlation with socioeconomic development: in high SDI regions, where advanced diagnostic tools are more accessible, the incidence gap between genders widens (34). This may reflect estrogen receptor imbalances and sex hormone interactions, which are exacerbated by socioeconomic factors (31). Notably, the SDI index also correlates with thyroid cancer outcomes: as economic development improves, incidence rates rise due to increased detection of indolent tumors, while mortality and DALYs decline—a pattern underscoring the dual role of medical progress in both overdiagnosis and effective disease management (31).

### 4.3 Comparative analysis at the national level

At the national level, in 2021, India, China, and the United States ranked among the top in terms of new cases, deaths, and DALYs due to thyroid cancer, primarily because of their large population bases, varying levels of economic development, and healthcare resource availability. Studies (35, 36) indicate that from 2006 to 2014, the ASR of thyroid cancer among women in India increased from 2.5 to 3.5 per 100,000 individuals (a 37% increase). Approximately 51% of thyroid cancer cases among Indian women may be overdiagnosed, with this proportion reaching 74% among women under 35 years of age, 50% among those aged 35 to 54 years, and 30% among those aged 55 to 64 years. In China, the incidence and mortality rates of thyroid cancer have shown an upward trend between 2005 and 2015, particularly in urban areas (27). In 2015, China reported approximately 200,700 newly diagnosed thyroid cancer cases, accounting for 34.23% of all new global cases (4). Additionally, the mortality rate of thyroid cancer in China was relatively low, with an estimated 7,900 deaths in 2015. The incidence rate in urban areas is higher than that in rural areas, and the standardized incidence rate in eastern regions is higher than that in central and western regions (4). In the United States, the incidence of thyroid cancer has also been rising since 1973, from 3.6 cases per 100,000 individuals to 8.7 in 2002 (37). In 2015, there were approximately 62,450 new cases of thyroid cancer and 1,950 deaths in the United States (38). The incidence of thyroid cancer in the United States significantly increased between 1992 and 2017, especially among women (39). Despite the rising incidence, the mortality rate of thyroid cancer has remained relatively stable, likely due to advancements in early diagnosis and treatment (38).

Countries such as Saudi Arabia, Libya, and Vietnam have experienced rapid increases in new cases, potentially linked to rapid economic and social development and increased diagnostic efforts (40). Conversely, Ethiopia, Zimbabwe, and Pakistan have higher mortality rates and DALYs due to thyroid cancer. According to a study on the cancer burden in Ethiopia published in 2022, the country reported 2,500 new cases of thyroid cancer in 2019, with an ASMR of 1090 per 100,000 population, 28,680 DALYs, and an ASDR of 50.7 per 100,000 population (41). In Zimbabwe and other sub-Saharan African countries, iodine deficiency is considered a significant factor contributing to thyroid cancer (42). Areas with iodine deficiency generally have higher incidence rates of thyroid cancer, while regions like Thailand and Sudan have experienced declines in thyroid cancer incidence due to increased iodine intake (42). Despite these challenges, progress is being made in the treatment and management of thyroid cancer globally, including the discovery of new therapies and targets, providing patients with more treatment options (42). However, the distribution of these advancements is uneven among countries, particularly in resource-limited countries or regions such as Ethiopia, Zimbabwe, and Pakistan, which still face significant challenges in healthcare resources, diagnostic technologies, and health insurance. Therefore, improving thyroid cancer management capabilities in these countries or regions requires multifaceted efforts, including improving iodine nutrition status, strengthening early screening and diagnostic capabilities, and providing more medical resources and support (43). These measures will help reduce thyroid cancer mortality and DALYs in these countries or regions, thereby improving patient quality of life and overall health status.

### 4.4 Future trend predictions and challenges

The incidence of thyroid cancer varies significantly across regions and periods. According to the predictions from Bayesian age-period-cohort models, the ASIR among women of child-bearing age has been increasing annually since 2021, while there have been no notable changes in ASMR and ASDR. This indicates that the incidence of thyroid cancer varies significantly across different geographical areas and time spans. For instance, in Canada, the ASIR of thyroid cancer among women has increased nearly six-fold over 43 years, from 3.9 per 100,000 individuals to 23.4 (13). This data aligns with the trends predicted by our models, further confirming the persistent upward trend in thyroid cancer incidence.

Upon a deeper analysis of these predictions, we can readily discern their complex public health implications. On one hand, the continuing rise in incidence necessitates intensified efforts in thyroid cancer prevention and control, particularly among high-risk populations. By implementing more precise early screening strategies and effective interventions, we aim to curb the disease at its early stages, thereby fundamentally reducing the overall incidence of thyroid cancer (44). On the other hand, the stable mortality and DALYs levels suggest that we should further optimize existing diagnostic and treatment pathways, enhance treatment efficiency and quality, and ensure that every thyroid cancer patient receives timely and appropriate care, ultimately maximizing survival time and improving quality of life (4, 45). However, this also raises concerns about over-diagnosis, which may lead to unnecessary treatment and psychological distress for patients (46). Therefore, we must carefully distinguish whether the observed trends genuinely reflect improvements in health outcomes or are merely artifacts of increased diagnostic scrutiny (47, 48).

To address these challenges, prevention and control measures must be strengthened, especially among high-risk populations (49). Identifying and targeting high-risk groups, such as those with genetic susceptibility or specific environmental exposures, can optimize the effectiveness of screening programs (49, 50). With the expansion of active surveillance, stricter biopsy criteria, and updated guidelines, the continuing upward trend in thyroid cancer incidence may be curbed in the future (26, 51, 52).

Furthermore, it is important to acknowledge the limitations of the Bayesian model employed. The accuracy of these predictions depends on the variables included and potential confounding factors not considered, thus introducing uncertainties into the predictions (53, 54). Therefore, continuous monitoring and validation of these models are crucial for refining future predictions and informing public health strategies (54).

### 4.5 Limitations and future directions of the study

Despite providing a comprehensive analysis of the global burden of thyroid cancer among women of child-bearing age, this study had certain limitations. First, while we identified epidemiological trends linked to SDI and diagnostic practices, the study does not provide etiological explanations for the observed disparities, such as molecular mechanisms or gene-environment interactions. Moreover, due to the GBD database’s classification at the organ-system level, we were unable to stratify results by thyroid cancer subtypes (e.g., papillary, follicular, medullary, anaplastic), which exhibit marked differences in clinical behavior and prognosis. This limitation may obscure important heterogeneity in disease burden and outcomes across subtypes.

The quality and completeness of the data may vary across regions, and diagnostic criteria and methods may change over time—factors that could introduce bias in cross-regional comparisons. Critically, these limitations underscore the need for standardized data collection protocols and longitudinal studies to validate causal inferences. Future research should integrate multidatabase approaches to enable subtype-specific analyses and explore molecular epidemiological factors driving subtype diversity, thereby refining prevention and treatment strategies.

To address these gaps, targeted prevention and control strategies should prioritize improving diagnostic capacity in low-SDI regions and integrating molecular epidemiology into future research designs, thereby reducing mortality and DALYs through more precise risk stratification.

## 5 Conclusions

The global burden of thyroid cancer among women of child-bearing age has undergone significant changes over the past 30 years, with a notable increase in incidence and slight decline in mortality and DALYs rates. Regional and national disparities, particularly the availability of national healthcare infrastructure for thyroid cancer screening and treatment, remain closely tied to SDI levels and directly shape the observed variations in outcomes. The accuracy and completeness of international cancer registries further influence our understanding of these disparities and the prioritization of global health interventions. As the global population ages and thyroid cancer incidence continues to rise, optimizing national healthcare systems—especially in low-SDI regions—through strengthened screening programs, equitable treatment access, and data transparency will be critical to reducing mortality and DALYs. Simultaneously, enhancing international data-sharing standards and collaboration is essential to address the evolving global burden of thyroid cancer and ensure equitable progress in prevention and care.

## Data Availability

The original contributions presented in the study are included in the article/**Supplementary Material**. Further inquiries can be directed to the corresponding author.

## References

[1] Li Y Piao J Li M. Secular trends in the epidemiologic patterns of thyroid cancer in China over three decades: an updated systematic analysis of global burden of disease study 2019 data. Front Endocrinol (Lausanne). (2021) 12:707233. doi: 10.3389/fendo.2021.707233 34526968 PMC8435774

[2] Kitahara CM Sosa JA. The changing incidence of thyroid cancer. Nat Rev Endocrinol. (2016) 12:646–53. doi: 10.1038/nrendo.2016.110 PMC10311569 27418023

[3] Tabatabaizadeh M Hasibi Taheri S Eydi M Shayestehpour M. The occurrence of Adrenocorticotropic hormone-independent Cushing's syndrome in a woman with the history of papillary thyroid carcinoma: a case report. J Med Case Rep. (2021) 15:113. doi: 10.1186/s13256-021-02684-x 33691778 PMC7948365

[4] Du L Zhao Z Zheng R Li H Zhang S Li R. Epidemiology of thyroid cancer: incidence and mortality in China, 2015. Front Oncol. (2020) 10:1702. doi: 10.3389/fonc.2020.01702 33240801 PMC7683719

[5] Boucai L Zafereo M Cabanillas ME. Thyroid cancer: A review. JAMA. (2024) 331:425–35. doi: 10.1001/jama.2023.26348 38319329

[6] Landa I Cabanillas ME. Genomic alterations in thyroid cancer: biological and clinical insights. Nat Rev Endocrinol. (2024) 20:93–110. doi: 10.1038/s41574-023-00920-6 38049644

[7] Vaccarella S Franceschi S Bray F Wild CP Plummer M Dal Maso L. Worldwide thyroid-cancer epidemic? The increasing impact of overdiagnosis. N Engl J Med. (2016) 375:614–7. doi: 10.1056/NEJMp1604412 27532827

[8] Lorenz K Schneider R Elwerr M. Thyroid carcinoma: do we need to treat men and women differently. Visc Med. (2020) 36:10–4. doi: 10.1159/000505496 PMC7036538 32110651

[9] Park J Kim K Lim DJ Bae JS Kim JS. Male sex is not an independent risk factor for recurrence of differentiated thyroid cancer: a propensity score-matching study. Sci Rep. (2021) 11:14908. doi: 10.1038/s41598-021-94461-5 34290341 PMC8295365

[10] Ding Y Zhong F. Effect of childbearing-age women's family status on the health status of three generations: evidence from China. Front Public Health. (2023) 11:1244581. doi: 10.3389/fpubh.2023.1244581 37780425 PMC10536147

[11] Eliassen FM Blåfjelldal V Helland T Hjorth CF Hølland K Lode L. Importance of endocrine treatment adherence and persistence in breast cancer survivorship: a systematic review. BMC Cancer. (2023) 23:625. doi: 10.1186/s12885-023-11122-8 37403065 PMC10320891

[12] Collaborators G2D. Global, regional, and national burden of diabetes from 1990 to 2021, with projections of prevalence to 2050: a systematic analysis for the Global Burden of Disease Study 2021. Lancet. (2023) 402:203–34. doi: 10.1016/S0140-6736(23)01301-6 PMC10364581 37356446

[13] Topstad D Dickinson JA. Thyroid cancer incidence in Canada: a national cancer registry analysis. CMAJ Open. (2017) 5:E612–612E616. doi: 10.9778/cmajo.20160162 PMC5621959 28807924

[14] Lee R Mason A members of the NTA Network. Is low fertility really a problem? Population aging, dependency, and consumption. Science. (2014) 346:229–34. doi: 10.1126/science.1250542 PMC4545628 25301626

[15] Scherer HC Fernandes PM Scheffel RS Zanella AB Maia AL Dora JM. Papillary thyroid microcarcinoma: insights from a cohort of 257 thyroidectomized patients. Horm Metab Res. (2023) 55:161–8. doi: 10.1055/a-2008-0824 36796412

[16] Lim H Devesa SS Sosa JA Check D Kitahara CM. Trends in thyroid cancer incidence and mortality in the United States, 1974-2013. JAMA. (2017) 317:1338–48. doi: 10.1001/jama.2017.2719 PMC8216772 28362912

[17] Chen AY Jemal A Ward EM. Increasing incidence of differentiated thyroid cancer in the United States, 1988-2005. Cancer. (2009) 115:3801–7. doi: 10.1002/cncr.24416 19598221

[18] Wu SS Lamarre ED Yalamanchali A Brauer PR Hong H Reddy CA. Association of treatment strategies and tumor characteristics with overall survival among patients with anaplastic thyroid cancer: A single-institution 21-year experience. JAMA Otolaryngol Head Neck Surg. (2023) 149:300–9. doi: 10.1001/jamaoto.2022.5045 PMC9912167 36757708

[19] Frasca F Piticchio T Le Moli R Tumino D Cannavò S Ruggeri RM. Early detection of suspicious lymph nodes in differentiated thyroid cancer. Expert Rev Endocrinol Metab. (2022) 17:447–54. doi: 10.1080/17446651.2022.2112176 35993330

[20] Shobab L Burman KD Wartofsky L. Sex differences in differentiated thyroid cancer. Thyroid. (2022) 32:224–35. doi: 10.1089/thy.2021.0361 34969307

[21] O'Grady TJ Rinaldi S Michels KA Adami HO Buring JE Chen Y. Association of hormonal and reproductive factors with differentiated thyroid cancer risk in women: a pooled prospective cohort analysis. Int J Epidemiol. (2024) 53:dyad172. doi: 10.1093/ije/dyad172 38110618 PMC10859160

[22] Kitahara CM Slettebø Daltveit D Ekbom A Engeland A Gissler M Glimelius I. Maternal health, pregnancy and offspring factors, and maternal thyroid cancer risk: A nordic population-based registry study. Am J Epidemiol. (2023) 192:70–83. doi: 10.1093/aje/kwac163 36130211 PMC10144719

[23] Chen DW Lang B McLeod D Newbold K Haymart MR. Thyroid cancer. Lancet. (2023) 401:1531–44. doi: 10.1016/S0140-6736(23)00020-X 37023783

[24] Stagnaro-Green A Dong A Stephenson MD. Universal screening for thyroid disease during pregnancy should be performed. Best Pract Res Clin Endocrinol Metab. (2020) 34:101320. doi: 10.1016/j.beem.2019.101320 31530447

[25] Nilubol N Zhang L Kebebew E. Multivariate analysis of the relationship between male sex, disease-specific survival, and features of tumor aggressiveness in thyroid cancer of follicular cell origin. Thyroid. (2013) 23:695–702. doi: 10.1089/thy.2012.0269 23194434 PMC3675841

[26] Gong Y Jiang Q Zhai M Tang T Liu S. Thyroid cancer trends in China and its comparative analysis with G20 countries: Projections for 2020-2040. J Glob Health. (2024) 14:4131. doi: 10.7189/jogh.14.04131 PMC11177899 38873786

[27] Cheng F Xiao J Shao C Huang F Wang L Ju Y. Burden of thyroid cancer from 1990 to 2019 and projections of incidence and mortality until 2039 in China: findings from global burden of disease study. Front Endocrinol (Lausanne). (2021) 12:738213. doi: 10.3389/fendo.2021.738213 34690931 PMC8527095

[28] Piticchio T Russ G Radzina M Frasca F Durante C Trimboli P. Head-to-head comparison of American, European, and Asian TIRADSs in thyroid nodule assessment: systematic review and meta-analysis. Eur Thyroid J. (2024) 13:e230242. doi: 10.1530/ETJ-23-0242 38417254 PMC10959032

[29] Shah BR Griffiths R Hall SF. Thyroid cancer incidence among Asian immigrants to Ontario, Canada: A population-based cohort study. Cancer. (2024) 123:3320–5. doi: 10.1002/cncr.30746 28440952

[30] Wang Q Huang H Zhao N Ni X Udelsman R Zhang Y. Phytoestrogens and thyroid cancer risk: a population-based case-control study in connecticut. Cancer Epidemiol Biomarkers Prev. (2020) 29(2):500–8. doi: 10.1158/1055-9965.EPI-19-0456 PMC7007342 31826911

[31] Zhou T Wang X Zhang J Zhou E Xu C Shen Y. Global burden of thyroid cancer from 1990 to 2021: a systematic analysis from the Global Burden of Disease Study 2021. J Hematol Oncol. (2024) 17:74. doi: 10.1186/s13045-024-01593-y 39192360 PMC11348565

[32] Collaboration GBoDC. Global, regional, and national cancer incidence, mortality, years of life lost, years lived with disability, and disability-adjusted life-years for 29 cancer groups, 1990 to 2016: A systematic analysis for the global burden of disease study. JAMA Oncol. (2018) 4:1553–68. doi: 10.1001/jamaoncol.2018.2706 PMC6248091 29860482

[33] Amendola S Piticchio T Scappaticcio L Sellasie SW Volpe S Le Moli R. Papillary thyroid carcinoma: ≤ 10 mm does not always mean pN0. A multicentric real-world study. Updates Surg. (2024) 76:1055–61. doi: 10.1007/s13304-024-01779-6 PMC11130044 38446376

[34] Nejadghaderi SA Moghaddam SS Azadnajafabad S Rezaei N Rezaei N Tavangar SM. Burden of thyroid cancer in North Africa and Middle East 1990-2019. Front Oncol. (2022) 12:955358. doi: 10.3389/fonc.2022.955358 36212501 PMC9538696

[35] Panato C Vaccarella S Dal Maso L Basu P Franceschi S Serraino D. Thyroid cancer incidence in India between 2006 and 2014 and impact of overdiagnosis. J Clin Endocrinol Metab. (2020) 105:2507–14. doi: 10.1210/clinem/dgaa192 PMC7947989 32297630

[36] Mathew IE Mathew A. Rising thyroid cancer incidence in southern India: an epidemic of overdiagnosis. J Endocr Soc. (2017) 1:480–7. doi: 10.1210/js.2017-00097 PMC5686600 29264503

[37] Pacini F. Changing natural history of differentiated thyroid cancer. Endocrine. (2012) 42:229–30. doi: 10.1007/s12020-012-9727-7 22736408

[38] Haugen BR Alexander EK Bible KC Doherty GM Mandel SJ Nikiforov YE. 2015 american thyroid association management guidelines for adult patients with thyroid nodules and differentiated thyroid cancer: the american thyroid association guidelines task force on thyroid nodules and differentiated thyroid cancer. Thyroid. (2016) 26:1–133. doi: 10.1089/thy.2015.0020 26462967 PMC4739132

[39] Cui Y Mubarik S Li R Nawsherwan Yu C. Trend dynamics of thyroid cancer incidence among China and the U.S. adult population from 1990 to 2017: a joinpoint and age-period-cohort analysis. BMC Public Health. (2021) 21:624. doi: 10.1186/s12889-021-10635-w 33789605 PMC8010947

[40] Rezaei Gazaki M Askari Shahi M Malboosbaf R Jambarsang S. Investigation of the global trend of thyroid cancer incidence and its relationship with the prevalence of type 2 diabetes: an application of longitudinal random effects regression model. Int J Cancer Manage. (2022) 15:e120720. doi: 10.5812/ijcm-120720

[41] Awedew AF Asefa Z Belay WB. National Burden and Trend of Cancer in Ethiopia, 2010-2019: a systemic analysis for Global burden of disease study. Sci Rep. (2022) 12:12736. doi: 10.1038/s41598-022-17128-9 35882895 PMC9325704

[42] Elhassan M Gismalla M Mohamed S Faggad A. Clinicopathological profile and management of thyroid carcinoma: a Sub-Saharan country experience. Thyroid Res. (2023) 16:35. doi: 10.1186/s13044-023-00173-5 37626413 PMC10463320

[43] Chagi N Bombil I Mannell A. The profile of thyroid cancer in patients undergoing thyroidectomy at Chris Hani Baragwanath Academic Hospital. S Afr J Surg. (2019) 57:55. doi: 10.17159/2078-5151/2019/v57n3a2928 31392867

[44] Krajewska J Kukulska A Oczko-Wojciechowska M Kotecka-Blicharz A Drosik-Rutowicz K Haras-Gil M. Early diagnosis of low-risk papillary thyroid cancer results rather in overtreatment than a better survival. Front Endocrinol (Lausanne). (2020) 11:571421. doi: 10.3389/fendo.2020.571421 33123090 PMC7573306

[45] Wang Q Zeng Z Nan J Zheng Y Liu H. Cause of death among patients with thyroid cancer: A population-based study. Front Oncol. (2022) 12:852347. doi: 10.3389/fonc.2022.852347 35359353 PMC8964038

[46] Hofmann B. Too much, too mild, too early: diagnosing the excessive expansion of diagnoses. Int J Gen Med. (2022) 15:6441–50. doi: 10.2147/IJGM.S368541 PMC9365059 35966506

[47] Maughan D James A. Diagnosis and treatment: Are psychiatrists choosing wisely. BJPsych Advances. (2017) 23:9–15. doi: 10.1192/apt.bp.115.015271

[48] Zaridze D Maximovitch D Smans M Stilidi I. Thyroid cancer overdiagnosis revisited. Cancer Epidemiol. (2021) 74:102014. doi: 10.1016/j.canep.2021.102014 34419801

[49] Vignali P Macerola E Poma AM Sparavelli R Basolo F. Indeterminate thyroid nodules: from cytology to molecular testing. Diagnostics. (2023) 13:3008. doi: 10.3390/diagnostics13183008 37761374 PMC10528553

[50] Eden K Mahon S Helfand M. Screening high-risk populations for thyroid cancer. Med Pediatr Oncol. (2001) 36:583–91. doi: 10.1002/mpo.1134 11340616

[51] Davies L Roman BR Fukushima M Ito Y Miyauchi A. Patient experience of thyroid cancer active surveillance in Japan. JAMA Otolaryngol Head Neck Surg. (2019) 145:363–70. doi: 10.1001/jamaoto.2018.4131 PMC6481594 30703198

[52] Zanocco KA Hershman JM Leung AM. Active surveillance of low-risk thyroid cancer. JAMA. (2019) 321:2020–1. doi: 10.1001/jama.2019.5350 31038662

[53] Tesema GA Tessema ZT Heritier S Stirling RG Earnest A. A systematic review of joint spatial and spatiotemporal models in health research. Int J Environ Res Public Health. (2023) 20:5295. doi: 10.3390/ijerph20075295 37047911 PMC10094468

[54] Li X Patel V Duan L Mikuliak J Basran J Osgood ND. Real-time epidemiology and acute care need monitoring and forecasting for COVID-19 via bayesian sequential monte carlo-leveraged transmission models. Int J Environ Res Public Health. (2024) 21:193. doi: 10.3390/ijerph21020193 38397684 PMC10888645

