# Supplementary material for: Global, regional, and national burden of thyroid cancer in women of child-bearing age, 1990 to 2021 and predictions to 2035: An analysis of the global burden of disease study 2021
Source: Front Endocrinol (Lausanne). 2025 Jun 27;16:1555841. doi: 10.3389/fendo.2025.1555841 (PMC12245710; doi:10.3389/fendo.2025.1555841)
Supplement: Supplementary file 1 [file DataSheet1.docx]

Supplementary Material

## 1. Supplementary Figures


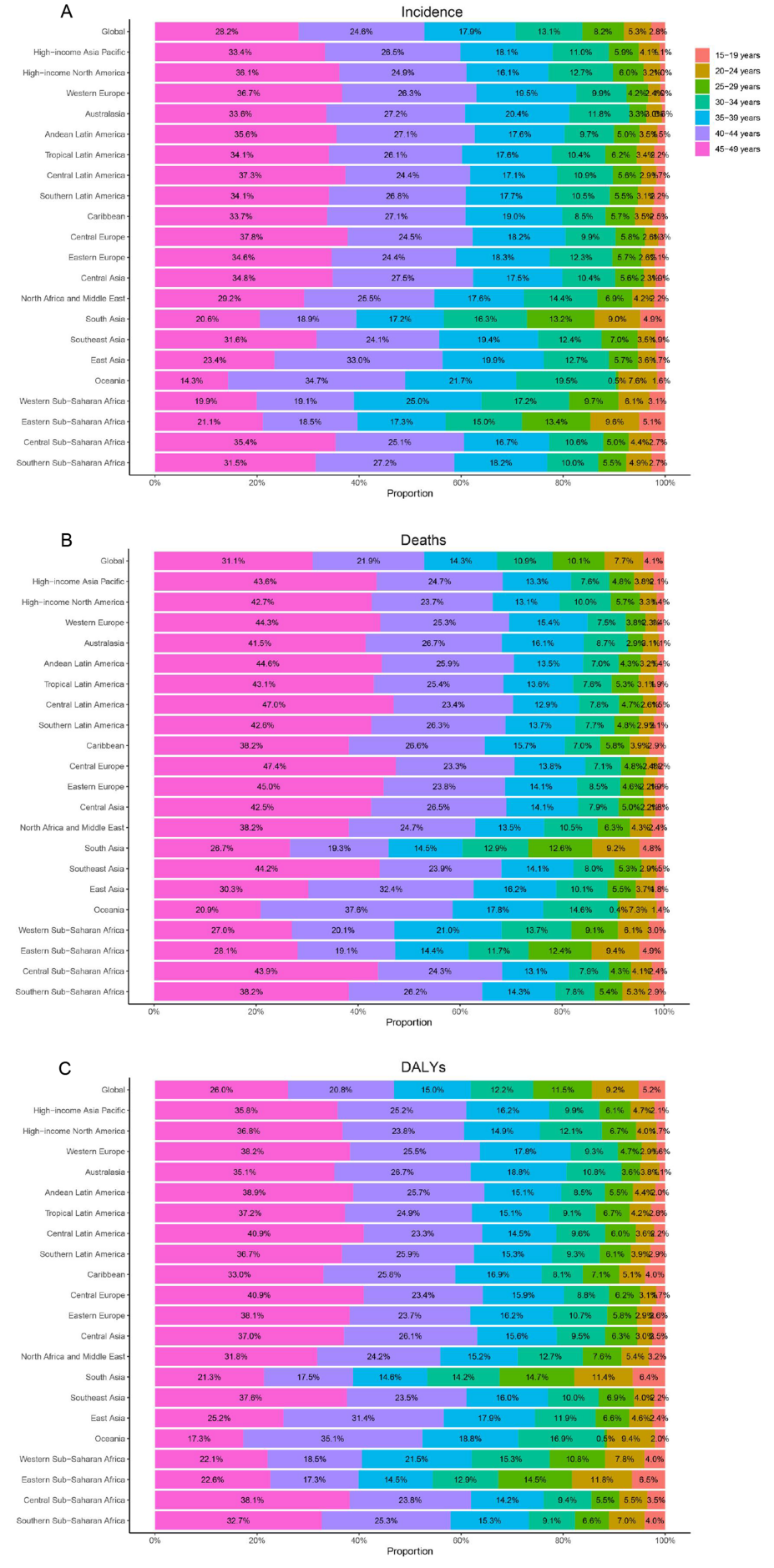


**Supplementary Figure 1.** Age-specific percentage of thyroid cancer among women of childbearing age incidence (A), deaths (B), and DALYs (C) rates in 2021. DALYs, disability-adjusted life years.


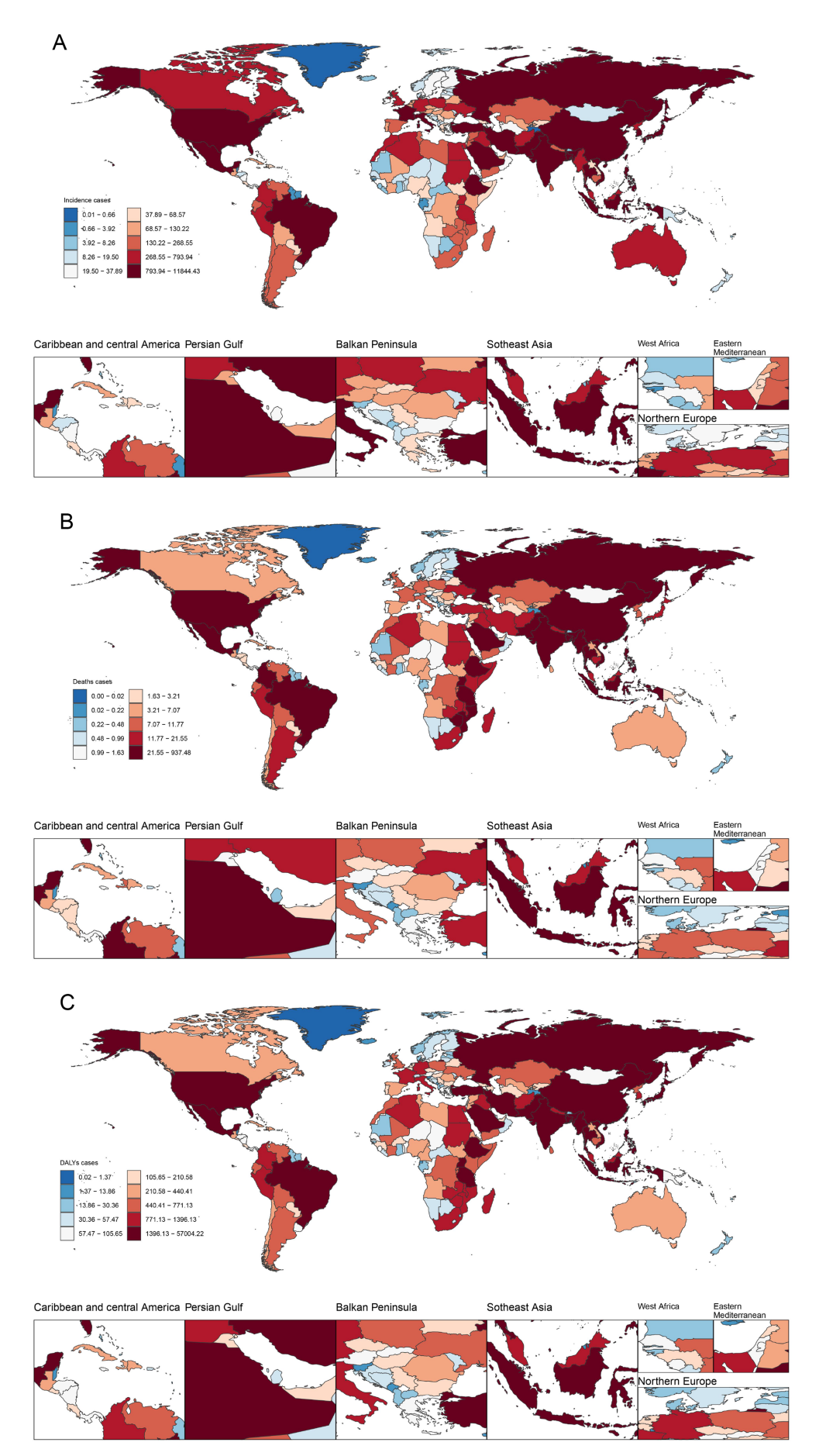


**Supplementary Figure 2.** The cases of incidence (A), deaths (B) , and DALYs (C) of thyroid cancer in women of childbearing age across 204 countries worldwide in 2021. DALYs, disability-adjusted life-years.

**
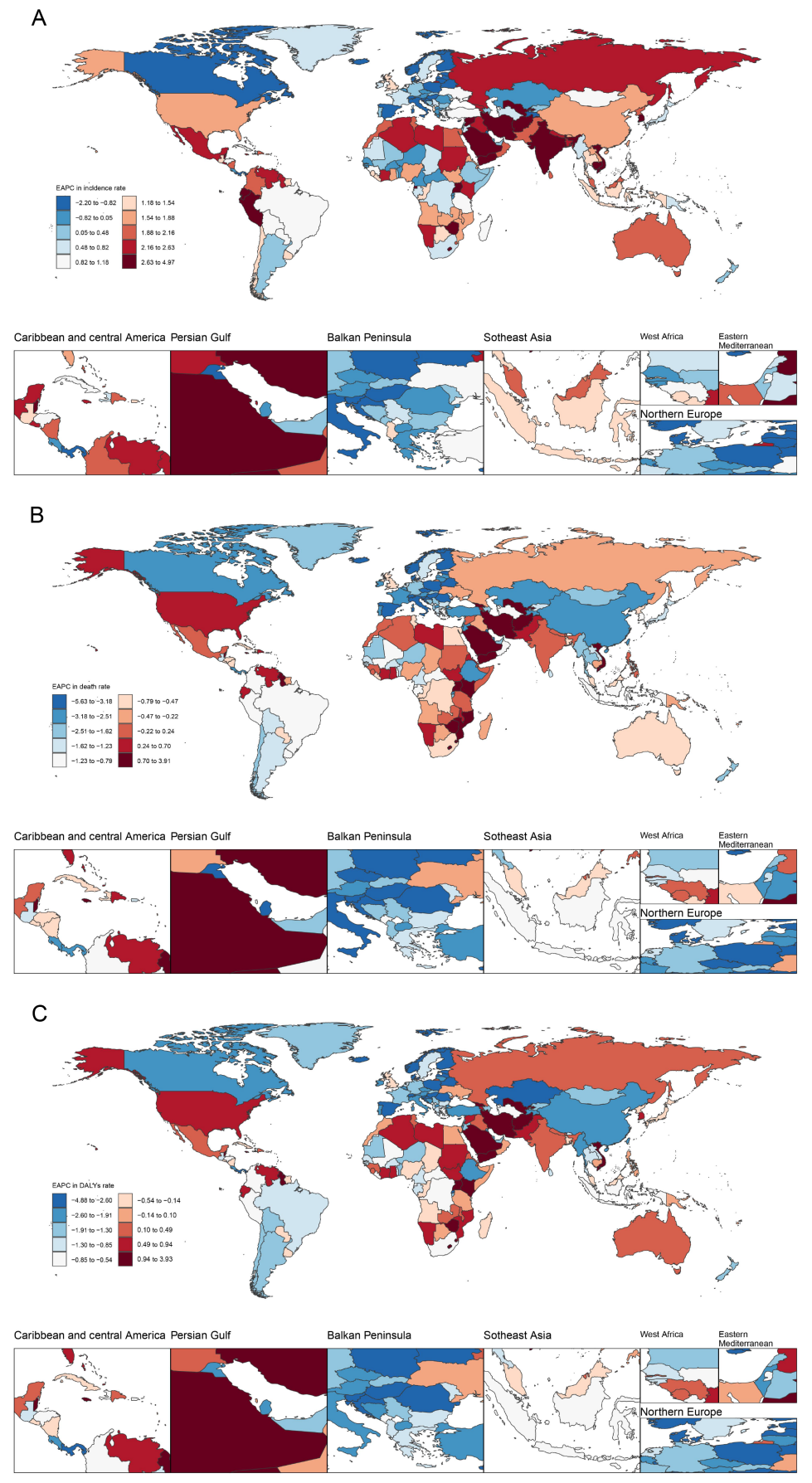
**

**Supplementary Figure 3.** The estimated annual percentage changes of ASIR (A), ASMR (B) , and ASDR (C) of thyroid cancer in women of childbearing age across 204 countries worldwide from 1990 to 2021. ASIR, age-standardized incidence rate; ASMR, age-standardized mortality rate; ASDR, age-standardized DALYs rate; DALYs, disability-adjusted life-years.

**
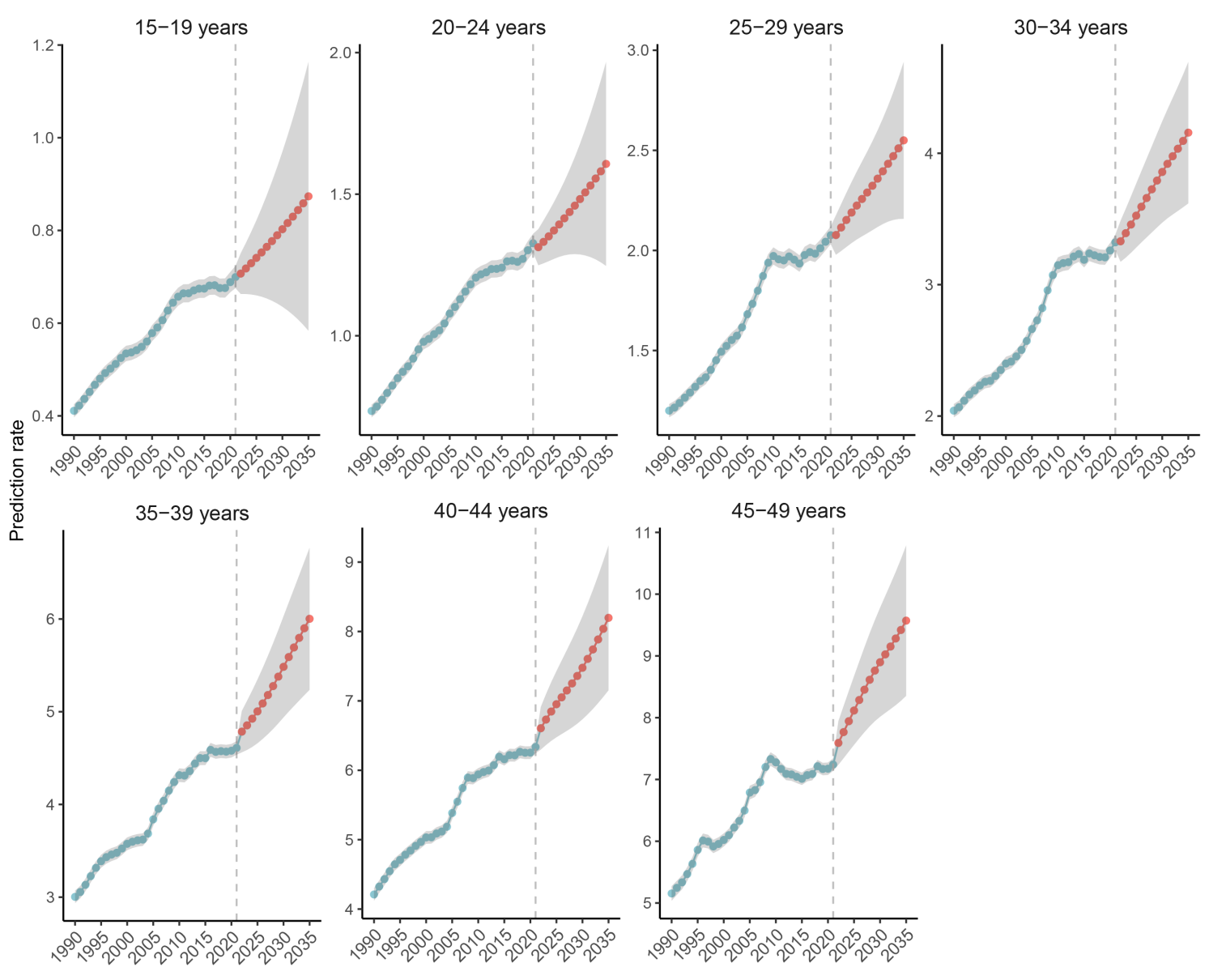
**

**Supplementary Figure 4.** Predictions of age-specific thyroid cancer incidence rates among women of childbearing age in 2035, based on global data.


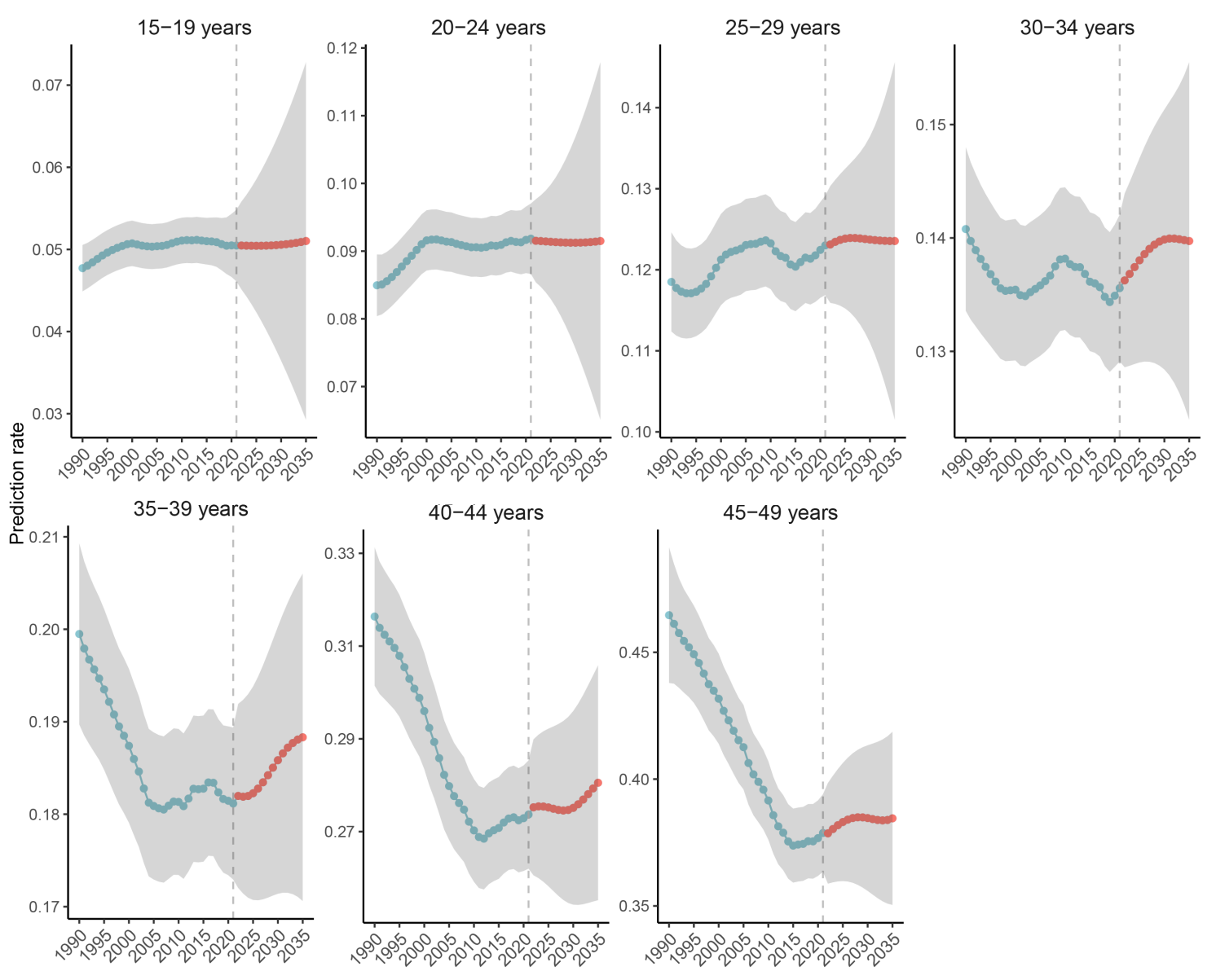


**Supplementary Figure 5.** Predictions of age-specific thyroid cancer mortality rates among women of childbearing age in 2035, based on global data.

**
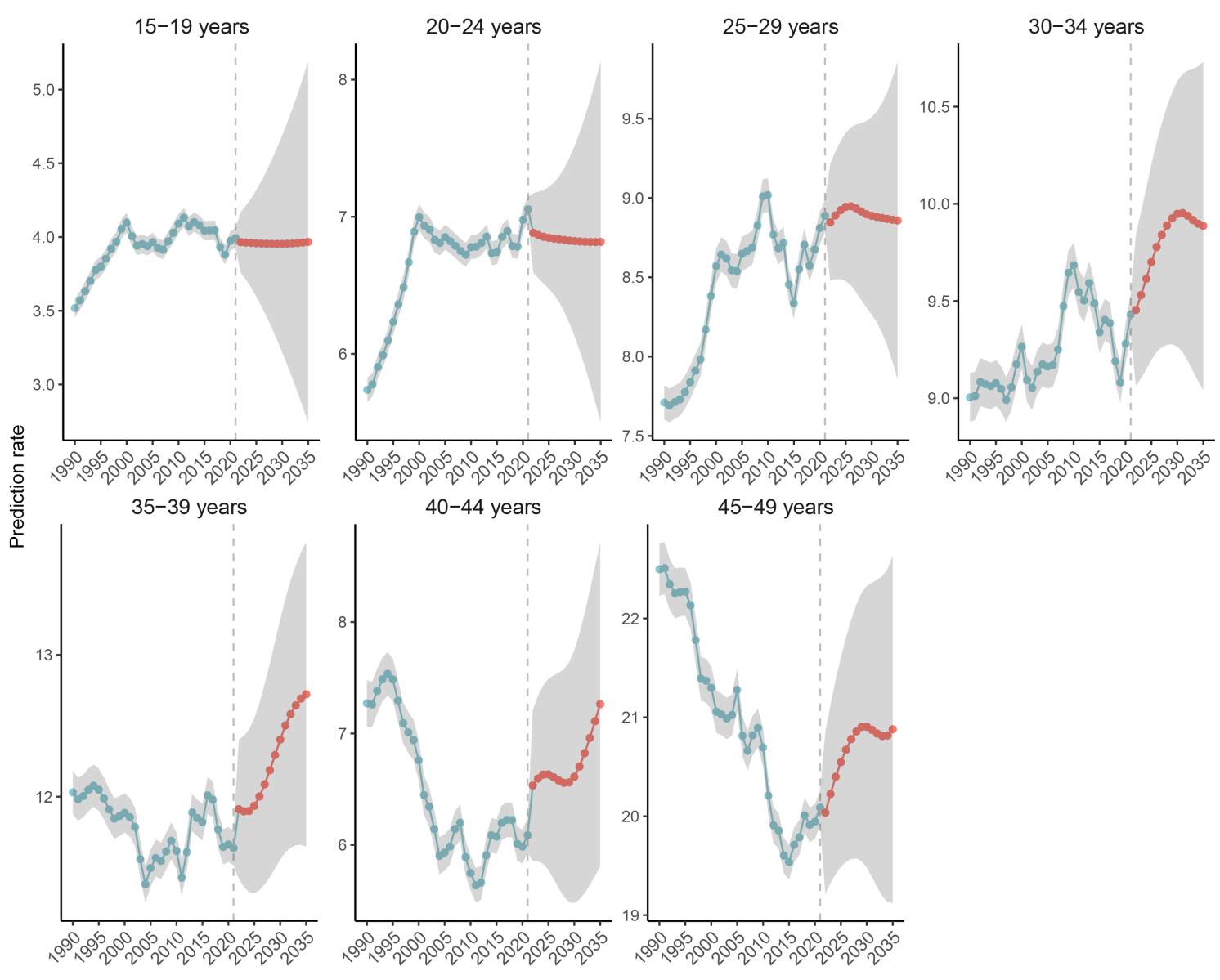
**

**Supplementary Figure 6.** Predictions of age-specific thyroid cancer DALYs rates among women of childbearing age in 2035, based on global data.

**2. Supplementary Tables**

**Supplementary Table 1.** Incidence, death, and DALYs of thyroid cancer in women of childbearing age (15-49 Years) in 204 Countries in 1990 and 2021, with estimated annual percentage changes from 1990 to 2021.

|  | **1990** | | | | | | **2021** | | | | | | **EAPC (1990-2021)** | | |
| --- | --- | --- | --- | --- | --- | --- | --- | --- | --- | --- | --- | --- | --- | --- | --- |
| **Characteristics** | **Incidence** cases  (95% UI) | **ASIR per 100-000**  (95% UI) | **Death** cases  (95% UI) | **ASMR per 100,000**  (95% UI) | **DALYs**  (95% UI) | **ASDR per 100,000**  (95% UI) | **Incidence** cases  (95% UI) | **ASIR per 100-000**  (95% UI) | **Death** cases  (95% UI) | **ASMR per 100,000**  (95% UI) | **DALYs**  (95% UI) | **ASDR per 100,000**  (95% UI) | **ASIR**  (95% CI) | **ASMR**  (95% CI) | **ASDR**  (95% CI) |
| Afghanistan | 37（8-112） | 2.01(0.43-6.02) | 5（1-14） | 0.25(0.06-0.74) | 263（58-796） | 13.71(3.01-41.41) | 284（87-615） | 4.87(1.50-10.44) | 21（7-43） | 0.36(0.11-0.72) | 1,249（394-2,550） | 20.83(6.60-41.89) | 3.30(3.01 to 3.58) | 1.48(1.36 to 1.59) | 1.62(1.50 to 1.75) |
| Albania | 10（6-17） | 1.42(0.78-2.39) | 1（0-1） | 0.12(0.07-0.19) | 47（28-76） | 6.68(3.92-10.64) | 13（6-23） | 1.91(0.91-3.57) | 0（0-1） | 0.06(0.03-0.11) | 27（14-50） | 4.12(2.03-7.61) | 1.54(1.10 to 1.99) | -1.73(-1.95 to -1.51) | -1.26(-1.49 to -1.03) |
| Algeria | 158（80-292） | 3.28(1.70-5.97) | 8（4-14） | 0.17(0.09-0.30) | 520（273-940） | 10.61(5.69-18.82) | 794（379-1,556） | 6.66(3.18-13.08) | 18（9-33） | 0.15(0.08-0.28) | 1,329（649-2,542） | 11.24(5.47-21.58) | 2.63(2.46 to 2.80) | -0.04(-0.20 to 0.12) | 0.51(0.36 to 0.66) |
| American Samoa | 0（0-0） | 1.92(0.98-3.49) | 0（0-0） | 0.15(0.08-0.25) | 1（0-1） | 8.30(4.43-14.53) | 0（0-1） | 3.70(1.87-6.65) | 0（0-0） | 0.18(0.10-0.31) | 1（1-2） | 11.09(5.81-19.21) | 2.04(1.70 to 2.38) | 0.80(0.59 to 1.00) | 0.99(0.77 to 1.21) |
| Andorra | 0（0-1） | 2.43(1.10-4.57) | 0（0-0） | 0.10(0.05-0.17) | 1（0-2） | 6.01(2.85-10.82) | 1（0-1） | 2.65(1.19-4.96) | 0（0-0） | 0.05(0.03-0.09) | 1（0-2） | 3.89(1.71-7.35) | 0.57(0.18 to 0.96) | -1.79(-1.96 to -1.62) | -1.13(-1.33 to -0.93) |
| Angola | 8（3-15） | 0.40(0.17-0.78) | 2（1-3） | 0.09(0.04-0.18) | 94（43-179） | 4.97(2.33-9.35) | 39（15-84） | 0.60(0.23-1.29) | 5（2-11） | 0.08(0.03-0.17) | 284（110-611） | 4.34(1.71-9.28) | 1.59(1.34 to 1.83) | -0.35(-0.54 to -0.16) | -0.21(-0.41 to -0.01) |
| Antigua and Barbuda | 0（0-1） | 2.35(1.73-3.11) | 0（0-0） | 0.18(0.16-0.22) | 2（1-2） | 10.60(8.83-12.57) | 1（1-1） | 3.32(2.45-4.43) | 0（0-0） | 0.16(0.14-0.19) | 3（2-3） | 9.68(8.05-11.77) | 1.90(1.32 to 2.49) | 0.26(-0.41 to 0.94) | 0.44(-0.21 to 1.10) |
| Argentina | 156（104-227） | 1.98(1.31-2.87) | 14（10-19） | 0.18(0.13-0.24) | 779（564-1,050） | 9.85(7.14-13.28) | 269（170-405） | 2.14(1.36-3.23) | 13（9-18） | 0.10(0.07-0.14) | 769（528-1,074） | 6.14(4.20-8.58) | 0.37(0.08 to 0.66) | -1.61(-1.96 to -1.26) | -1.34(-1.68 to -1.00) |
| Armenia | 14（9-22） | 1.83(1.18-2.70) | 1（1-1） | 0.14(0.10-0.19) | 60（41-85） | 7.76(5.34-10.77) | 25（16-37） | 2.93(1.86-4.34) | 1（1-1） | 0.13(0.09-0.18) | 64（44-91） | 7.71(5.24-10.92) | 2.33(1.88 to 2.77) | 0.37(-0.06 to 0.81) | 0.56(0.13 to 1.00) |
| Australia | 135（91-195） | 2.95(1.97-4.26) | 3（2-4） | 0.07(0.05-0.09) | 237（160-340） | 5.22(3.52-7.47) | 299（194-439） | 4.34(2.81-6.39) | 3（2-4） | 0.05(0.03-0.06) | 308（198-469） | 4.48(2.87-6.85) | 1.91(0.88 to 2.95) | -0.77(-1.51 to -0.03) | 0.19(-0.66 to 1.04) |
| Austria | 103（68-149） | 4.94(3.30-7.18) | 3（2-4） | 0.16(0.12-0.21) | 214（152-295） | 10.31(7.29-14.22) | 86（56-128） | 3.62(2.34-5.42) | 1（1-2） | 0.05(0.04-0.07) | 106（68-157） | 4.46(2.84-6.69) | -0.64(-1.00 to -0.28) | -3.12(-3.28 to -2.96) | -2.31(-2.50 to -2.12) |
| Azerbaijan | 17（9-29） | 1.12(0.61-1.92) | 2（1-3） | 0.12(0.07-0.19) | 97（54-160） | 6.45(3.68-10.42) | 34（17-63） | 1.17(0.59-2.15) | 2（1-4） | 0.07(0.04-0.12) | 120（64-212） | 4.14(2.21-7.32) | 0.17(-0.16 to 0.49) | -1.80(-1.98 to -1.63) | -1.62(-1.81 to -1.43) |
| Bahrain | 5（2-8） | 4.77(2.37-8.52) | 0（0-0） | 0.21(0.11-0.34) | 12（6-21） | 12.91(6.77-21.98) | 29（14-53） | 8.37(4.14-15.44) | 0（0-1） | 0.14(0.07-0.24) | 38（19-69） | 11.27(5.66-20.32) | 2.37(2.13 to 2.60) | -0.95(-1.15 to -0.75) | -0.02(-0.23 to 0.18) |
| Bangladesh | 329（148-619） | 1.51(0.70-2.80) | 59（28-105） | 0.28(0.14-0.49) | 3,547（1,663-6,375） | 15.87(7.60-28.16) | 1,338（515-3,411） | 2.94(1.14-7.45) | 96（40-234） | 0.21(0.09-0.51) | 6,010（2,407-14,936） | 13.16(5.31-32.47) | 2.54(2.31 to 2.78) | -0.63(-0.81 to -0.45) | -0.34(-0.54 to -0.13) |
| Barbados | 1（1-2） | 2.12(1.57-2.78) | 0（0-0） | 0.16(0.14-0.19) | 6（5-7） | 9.36(7.84-11.08) | 3（2-4） | 3.11(2.07-4.51) | 0（0-0） | 0.14(0.11-0.19) | 7（5-10） | 8.80(6.37-11.95) | 1.31(1.01 to 1.63) | -0.22(-0.58 to 0.14) | -0.04(-0.40 to 0.32) |
| Belarus | 99（63-148） | 3.82(2.43-5.73) | 4（3-6） | 0.18(0.12-0.24) | 282（189-407） | 11.03(7.41-15.89) | 92（54-142） | 3.40(2.00-5.28) | 2（1-3） | 0.08(0.05-0.12) | 155（94-242） | 5.76(3.48-9.03) | -1.23(-2.02 to -0.44) | -3.44(-4.27 to -2.60) | -3.11(-3.94 to -2.27) |
| Belgium | 68（45-99） | 2.65(1.74-3.86) | 3（2-4） | 0.12(0.09-0.17) | 188（136-261） | 7.40(5.34-10.25) | 74（48-110） | 2.53(1.63-3.78) | 2（1-2） | 0.06(0.04-0.08) | 117（76-168） | 3.99(2.59-5.79) | -0.08(-0.78 to 0.62) | -2.58(-3.11 to -2.05) | -1.99(-2.57 to -1.41) |
| Belize | 0（0-0） | 0.77(0.57-1.01) | 0（0-0） | 0.08(0.07-0.10) | 1（1-2） | 4.58(3.85-5.39) | 2（1-2） | 1.55(1.13-2.09) | 0（0-0） | 0.11(0.09-0.13) | 7（5-8） | 6.08(4.92-7.44) | 2.71(2.38 to 3.05) | 1.13(0.85 to 1.41) | 1.29(1.01 to 1.56) |
| Benin | 3（1-6） | 0.30(0.14-0.63) | 1（0-1） | 0.06(0.03-0.12) | 29（13-60） | 3.17(1.46-6.37) | 10（4-21） | 0.35(0.15-0.76) | 1（0-2） | 0.04(0.02-0.09) | 65（28-137） | 2.36(1.02-4.96) | 0.03(-0.15 to 0.22) | -1.46(-1.67 to -1.26) | -1.36(-1.57 to -1.15) |
| Bermuda | 1（0-1） | 2.60(1.64-3.91) | 0（0-0） | 0.15(0.10-0.20) | 2（1-2） | 8.76(5.95-12.22) | 1（0-1） | 4.26(2.55-6.69) | 0（0-0） | 0.10(0.06-0.14) | 1（1-2） | 6.93(4.33-10.75) | 1.42(1.01 to 1.83) | -1.62(-2.28 to -0.95) | -0.91(-1.53 to -0.29) |
| Bhutan | 2（1-4） | 1.44(0.60-2.75) | 0（0-1） | 0.26(0.12-0.49) | 19（8-37） | 15.05(6.56-28.08) | 6（2-14） | 2.80(1.07-6.91) | 0（0-1） | 0.22(0.09-0.52) | 27（10-67） | 13.21(5.19-32.59) | 1.85(1.74 to 1.97) | -1.03(-1.19 to -0.88) | -0.85(-1.01 to -0.69) |
| Bolivia | 29（13-53） | 2.24(1.01-4.02) | 6（3-10） | 0.45(0.21-0.78) | 309（141-545） | 23.58(10.85-41.24) | 100（48-187） | 3.37(1.61-6.26) | 9（5-17） | 0.32(0.16-0.56) | 519（255-933） | 17.46(8.62-31.26) | 1.08(0.97 to 1.19) | -1.42(-1.56 to -1.28) | -1.30(-1.44 to -1.17) |
| Bosnia and Herzegovina | 18（12-26） | 1.59(1.04-2.31) | 1（1-2） | 0.11(0.08-0.15) | 72（50-99） | 6.40(4.42-8.84) | 15（9-25） | 1.72(1.03-2.78) | 1（0-1） | 0.06(0.04-0.09) | 33（21-52） | 3.75(2.35-5.91) | 0.40(0.13 to 0.68) | -2.34(-2.61 to -2.07) | -1.91(-2.15 to -1.66) |
| Botswana | 2（1-4） | 0.75(0.30-1.70) | 0（0-1） | 0.13(0.05-0.27) | 16（6-36） | 6.60(2.70-14.41) | 6（2-14） | 0.87(0.31-2.09) | 1（0-1） | 0.09(0.04-0.21) | 33（12-78） | 4.99(1.91-11.83) | 1.37(0.52 to 2.23) | -0.30(-0.97 to 0.38) | -0.14(-0.82 to 0.55) |
| Brazil | 359（314-413） | 1.07(0.93-1.22) | 40（36-45） | 0.12(0.11-0.14) | 2,222（1,983-2,500） | 6.61(5.91-7.42) | 1,036（911-1,193） | 1.62(1.42-1.86) | 60（54-68） | 0.09(0.08-0.11) | 3,505（3,095-3,989） | 5.49(4.85-6.26) | 1.01(0.69 to 1.33) | -1.12(-1.32 to -0.91) | -0.88(-1.11 to -0.66) |
| Brunei | 2（1-4） | 3.71(1.73-6.84) | 0（0-0） | 0.21(0.11-0.38) | 7（3-12） | 12.46(6.14-22.18) | 6（3-10） | 4.51(2.39-7.75) | 0（0-0） | 0.15(0.09-0.24) | 13（7-21） | 9.51(5.30-15.89) | 1.63(1.28 to 1.97) | -0.12(-0.57 to 0.34) | 0.18(-0.24 to 0.59) |
| Bulgaria | 39（27-57） | 1.70(1.14-2.45) | 4（3-5） | 0.17(0.12-0.22) | 209（151-283） | 9.11(6.53-12.37) | 34（22-51） | 1.75(1.11-2.66) | 2（1-3） | 0.11(0.07-0.15) | 117（81-163） | 6.15(4.25-8.66) | 0.15(-0.18 to 0.47) | -1.37(-1.60 to -1.13) | -1.30(-1.55 to -1.05) |
| Burkina Faso | 8（3-16） | 0.42(0.18-0.86) | 1（1-3） | 0.08(0.03-0.16) | 77（33-157） | 4.25(1.84-8.58) | 19（8-40） | 0.42(0.18-0.84) | 3（1-5） | 0.06(0.02-0.11) | 146（63-290） | 3.11(1.37-6.13) | -0.13(-0.27 to 0.01) | -1.25(-1.44 to -1.06) | -1.17(-1.37 to -0.97) |
| Burundi | 23（10-45） | 2.00(0.89-3.88) | 5（2-9） | 0.45(0.21-0.85) | 296（134-562） | 25.51(11.77-48.19) | 56（24-120） | 1.98(0.85-4.20) | 8（4-18） | 0.31(0.14-0.66) | 518（225-1,123） | 17.82(7.85-38.78) | -0.38(-0.59 to -0.18) | -1.68(-1.89 to -1.47) | -1.57(-1.77 to -1.36) |
| Cambodia | 37（16-70） | 1.78(0.79-3.28) | 6（3-11） | 0.30(0.14-0.53) | 331（149-595） | 15.95(7.36-28.29) | 177（84-345） | 4.13(1.96-8.03) | 12（6-22） | 0.29(0.15-0.53) | 697（341-1,300） | 16.48(8.11-30.59) | 2.65(2.50 to 2.81) | -0.33(-0.51 to -0.14) | -0.13(-0.32 to 0.05) |
| Cameroon | 10（5-21） | 0.52(0.23-1.01) | 2（1-3） | 0.09(0.04-0.17) | 98（45-189） | 4.90(2.23-9.24) | 38（16-76） | 0.57(0.24-1.11) | 4（2-8） | 0.06(0.03-0.12) | 242（105-468） | 3.56(1.56-6.83) | -0.01(-0.19 to 0.17) | -1.38(-1.50 to -1.26) | -1.28(-1.40 to -1.16) |
| Canada | 304（202-440） | 3.93(2.61-5.67) | 8（6-10） | 0.10(0.07-0.13) | 538（368-742） | 7.02(4.81-9.67) | 317（204-473） | 3.36(2.15-5.01) | 5（3-6） | 0.05(0.04-0.07) | 396（250-602） | 4.20(2.64-6.42) | -1.08(-1.51 to -0.65) | -2.84(-3.27 to -2.41) | -2.28(-2.71 to -1.84) |
| Cape Verde | 0（0-0） | 0.17(0.07-0.34) | 0（0-0） | 0.02(0.01-0.04) | 1（0-1） | 1.07(0.49-2.12) | 1（0-2） | 0.77(0.18-1.63) | 0（0-0） | 0.04(0.01-0.09) | 4（1-8） | 2.67(0.63-5.51) | 4.32(2.99 to 5.66) | 1.82(0.53 to 3.12) | 2.07(0.75 to 3.41) |
| Central African Republic | 2（1-4） | 0.45(0.21-0.84) | 1（0-1） | 0.12(0.06-0.22) | 32（15-59） | 6.20(3.01-11.27) | 5（2-10） | 0.43(0.18-0.87) | 1（0-2） | 0.09(0.04-0.19) | 57（24-116） | 4.95(2.07-9.87) | -0.28(-0.37 to -0.18) | -0.89(-0.98 to -0.79) | -0.84(-0.93 to -0.74) |
| Chad | 3（1-7） | 0.27(0.12-0.57) | 1（0-1） | 0.06(0.03-0.11) | 34（15-70） | 3.04(1.37-6.14) | 10（5-20） | 0.33(0.15-0.64) | 2（1-3） | 0.05(0.03-0.10) | 88（42-165） | 2.84(1.36-5.31) | 0.58(0.49 to 0.68) | -0.37(-0.49 to -0.26) | -0.31(-0.41 to -0.20) |
| Chile | 74（48-109） | 2.26(1.47-3.34) | 6（4-8） | 0.19(0.13-0.25) | 338（237-467） | 10.37(7.28-14.25) | 158（102-236） | 3.10(1.99-4.66) | 5（3-7） | 0.09(0.07-0.13) | 318（215-457） | 6.25(4.22-8.99) | 1.32(1.00 to 1.63) | -2.01(-2.42 to -1.60) | -1.41(-1.82 to -1.00) |
| China | 4,324（2,991-5,696） | 1.53(1.06-2.01) | 397（276-523） | 0.14(0.10-0.19) | 23,020（15,988-30,733） | 8.10(5.63-10.77) | 10,017（7,004-15,723） | 2.65(1.85-4.18) | 251（175-389） | 0.07(0.05-0.10) | 17,517（11,855-27,200） | 4.68(3.16-7.31) | 1.80(1.65 to 1.95) | -2.91(-3.07 to -2.76) | -2.10(-2.26 to -1.95) |
| Colombia | 165（111-237） | 2.25(1.52-3.20) | 17（13-22） | 0.24(0.18-0.31) | 977（705-1,301） | 13.30(9.70-17.55) | 532（327-821） | 4.00(2.46-6.17) | 23（15-33） | 0.17(0.12-0.25) | 1,396（928-2,071） | 10.50(6.99-15.57) | 1.89(1.59 to 2.18) | -1.08(-1.48 to -0.68) | -0.76(-1.15 to -0.37) |
| Comoros | 2（1-4） | 1.89(0.79-4.06) | 0（0-1） | 0.35(0.16-0.72) | 19（8-41） | 20.11(8.68-41.90) | 5（2-12） | 2.77(1.07-6.39) | 1（0-1） | 0.34(0.14-0.75) | 38（15-85） | 19.74(7.86-44.19) | 0.88(0.47 to 1.30) | -0.61(-1.06 to -0.16) | -0.55(-1.04 to -0.05) |
| Congo | 3（1-5） | 0.60(0.27-1.13) | 1（0-1） | 0.13(0.06-0.23) | 28（13-52） | 6.58(3.05-12.02) | 13（5-27） | 0.96(0.38-1.99) | 2（1-3） | 0.11(0.05-0.23) | 82（35-165） | 6.22(2.62-12.41) | 1.46(1.22 to 1.71) | -0.52(-0.73 to -0.31) | -0.36(-0.58 to -0.15) |
| Cook Islands | 0（0-0） | 0.27(0.12-0.55) | 0（0-0） | 0.02(0.01-0.03) | 0（0-0） | 0.95(0.45-1.85) | 0（0-0） | 0.63(0.27-1.22) | 0（0-0） | 0.01(0.01-0.03) | 0（0-0） | 1.09(0.48-2.06) | 3.19(2.65 to 3.74) | 0.25(-0.20 to 0.69) | 0.96(0.51 to 1.42) |
| Costa Rica | 23（15-33） | 3.38(2.24-4.86) | 1（1-2） | 0.22(0.16-0.28) | 84（61-114） | 12.59(9.14-16.92) | 46（29-70） | 3.43(2.16-5.21) | 2（1-2） | 0.13(0.09-0.18) | 107（72-157） | 7.99(5.34-11.65) | -0.78(-1.36 to -0.18) | -2.54(-3.13 to -1.94) | -2.34(-2.95 to -1.73) |
| Cote d'Ivoire | 23（11-44） | 0.96(0.47-1.81) | 4（2-7） | 0.17(0.08-0.29) | 228（112-423） | 9.41(4.73-16.99) | 102（46-201） | 1.64(0.75-3.18) | 11（5-20） | 0.18(0.09-0.33) | 651（303-1,267） | 10.39(4.94-19.86) | 2.27(2.03 to 2.52) | 0.68(0.52 to 0.84) | 0.82(0.66 to 0.98) |
| Croatia | 55（36-80） | 4.22(2.77-6.17) | 2（1-3） | 0.15(0.11-0.20) | 121（85-168） | 9.42(6.59-13.07) | 34（21-51） | 3.10(1.93-4.64) | 1（0-1） | 0.06(0.04-0.08) | 47（30-71） | 4.28(2.68-6.46) | -0.95(-1.25 to -0.66) | -3.29(-3.54 to -3.04) | -2.63(-2.90 to -2.37) |
| Cuba | 63（41-92） | 2.24(1.46-3.26) | 4（3-5） | 0.14(0.10-0.18) | 230（161-312） | 8.07(5.69-10.93) | 95（61-146） | 3.30(2.08-5.07) | 4（2-5） | 0.12(0.08-0.16) | 217（143-312） | 7.44(4.88-10.79) | 1.12(0.64 to 1.60) | -0.65(-1.22 to -0.08) | -0.38(-0.95 to 0.19) |
| Cyprus | 5（2-9） | 2.44(1.18-4.52) | 0（0-1） | 0.16(0.08-0.27) | 18（9-33） | 9.07(4.55-16.18) | 8（4-16） | 1.73(0.80-3.60) | 0（0-0） | 0.04(0.02-0.08) | 12（5-26） | 2.71(1.23-5.98) | -1.73(-2.48 to -0.98) | -5.58(-6.18 to -4.98) | -4.83(-5.46 to -4.20) |
| Czech Republic | 146（98-210） | 5.05(3.39-7.31) | 5（4-6） | 0.17(0.13-0.22) | 316（224-434） | 11.05(7.79-15.22) | 130（82-197） | 4.22(2.63-6.40) | 2（1-3） | 0.06(0.04-0.08) | 158（101-236） | 5.15(3.23-7.80) | -0.64(-1.17 to -0.11) | -3.21(-3.50 to -2.92) | -2.41(-2.76 to -2.06) |
| Democratic Republic of the Congo | 27（11-55） | 0.39(0.16-0.79) | 6（2-11） | 0.08(0.04-0.16) | 306（131-600） | 4.38(1.91-8.53) | 85（31-190） | 0.48(0.18-1.08) | 12（4-26） | 0.07(0.03-0.15) | 656（244-1,446） | 3.70(1.39-8.15) | 0.58(0.16 to 1.00) | -0.64(-0.89 to -0.39) | -0.60(-0.86 to -0.34) |
| Denmark | 25（17-36） | 1.72(1.17-2.47) | 1（1-2） | 0.10(0.08-0.13) | 85（62-113） | 5.90(4.27-7.86) | 13（9-20） | 0.91(0.58-1.37) | 0（0-1） | 0.03(0.02-0.04) | 27（18-40） | 1.85(1.20-2.70) | -1.72(-2.60 to -0.82) | -3.98(-4.72 to -3.23) | -3.60(-4.38 to -2.82) |
| Djibouti | 1（0-2） | 1.33(0.53-2.90) | 0（0-0） | 0.24(0.10-0.50) | 11（4-25） | 13.03(5.35-28.41) | 7（2-17） | 2.14(0.75-5.40) | 1（0-2） | 0.25(0.09-0.60) | 45（16-111） | 14.05(5.03-34.87) | 1.63(1.48 to 1.78) | 0.17(-0.05 to 0.39) | 0.28(0.05 to 0.51) |
| Dominica | 0（0-0） | 1.01(0.58-1.65) | 0（0-0） | 0.11(0.07-0.17) | 1（1-1） | 6.02(3.62-9.51) | 0（0-0） | 1.35(0.68-2.39) | 0（0-0） | 0.10(0.05-0.17) | 1（1-2） | 5.90(3.01-10.17) | 1.24(0.97 to 1.51) | 0.15(-0.21 to 0.51) | 0.33(-0.03 to 0.69) |
| Dominican Republic | 17（10-29） | 1.11(0.63-1.89) | 2（1-4） | 0.15(0.09-0.24) | 123（74-203） | 7.99(4.87-13.01) | 53（26-98） | 1.89(0.94-3.47) | 4（2-7） | 0.14(0.07-0.25) | 228（117-405） | 8.10(4.17-14.34) | 2.02(1.80 to 2.23) | 0.33(0.13 to 0.54) | 0.49(0.29 to 0.68) |
| Ecuador | 31（21-45） | 1.46(0.98-2.12) | 4（3-5） | 0.19(0.14-0.26) | 224（161-304） | 10.57(7.68-14.25) | 180（106-287） | 3.91(2.30-6.22) | 11（7-16） | 0.23(0.15-0.35) | 618（385-942） | 13.41(8.36-20.42) | 3.19(2.45 to 3.94) | 0.68(0.16 to 1.20) | 0.84(0.31 to 1.38) |
| Egypt | 163（83-283） | 1.42(0.73-2.45) | 11（6-18） | 0.10(0.06-0.16) | 664（352-1,097） | 5.75(3.10-9.39) | 645（328-1,133） | 2.62(1.34-4.60) | 19（11-33） | 0.08(0.05-0.13) | 1,310（696-2,285） | 5.35(2.86-9.28) | 1.97(1.86 to 2.08) | -0.47(-0.61 to -0.34) | -0.02(-0.15 to 0.11) |
| El Salvador | 16（11-23） | 1.58(1.07-2.23) | 2（2-3） | 0.22(0.16-0.29) | 119（85-159） | 11.52(8.21-15.22) | 59（37-90） | 3.37(2.12-5.14) | 3（2-5） | 0.18(0.12-0.26) | 188（125-271） | 10.74(7.16-15.49) | 2.52(2.30 to 2.74) | -0.55(-0.80 to -0.30) | -0.20(-0.41 to 0.02) |
| Equatorial Guinea | 0（0-1） | 0.40(0.18-0.78) | 0（0-0） | 0.10(0.05-0.19) | 4（2-8） | 5.21(2.35-10.08) | 3（1-7） | 0.94(0.32-2.11) | 0（0-1） | 0.08(0.03-0.17) | 14（5-30） | 4.48(1.62-9.92) | 3.16(2.91 to 3.40) | -0.83(-1.00 to -0.66) | -0.55(-0.72 to -0.39) |
| Eritrea | 10（5-19） | 1.55(0.75-2.83) | 2（1-4） | 0.36(0.19-0.63) | 134（67-241） | 19.56(10.00-34.60) | 37（15-76） | 2.41(1.02-4.98) | 6（2-11） | 0.37(0.17-0.74) | 319（138-650） | 20.84(9.13-41.98) | 1.59(1.47 to 1.71) | 0.31(0.20 to 0.43) | 0.42(0.31 to 0.54) |
| Estonia | 19（12-28） | 4.59(2.95-6.88) | 1（1-1） | 0.20(0.14-0.27) | 51（35-72） | 12.63(8.66-18.00) | 10（7-15） | 2.99(1.90-4.47) | 0（0-0） | 0.06(0.04-0.09) | 16（10-23） | 4.62(2.98-6.77) | -0.93(-1.82 to -0.03) | -3.72(-4.46 to -2.97) | -3.07(-3.85 to -2.30) |
| Ethiopia | 448（236-762） | 4.47(2.38-7.57) | 111（60-184） | 1.13(0.63-1.87) | 6,477（3,505-10,795） | 63.16(34.63-104.89) | 1,371（708-2,909） | 5.45(2.84-11.58) | 155（85-322） | 0.63(0.35-1.32) | 9,581（5,236-19,917） | 37.27(20.52-77.83) | 0.10(-0.21 to 0.41) | -2.51(-2.77 to -2.25) | -2.34(-2.61 to -2.08) |
| Federated States of Micronesia | 0（0-0） | 0.78(0.36-1.46) | 0（0-0） | 0.10(0.05-0.19) | 1（1-2） | 5.74(2.74-10.61) | 0（0-1） | 1.39(0.65-2.74) | 0（0-0） | 0.10(0.05-0.18) | 1（1-3） | 5.73(2.91-10.68) | 1.94(1.81 to 2.07) | -0.17(-0.21 to -0.13) | 0.03(-0.02 to 0.07) |
| Fiji | 5（2-9） | 2.62(1.31-4.82) | 0（0-1） | 0.25(0.14-0.44) | 25（13-44） | 14.32(7.74-25.14) | 8（4-16） | 3.55(1.64-7.02) | 1（0-1） | 0.22(0.11-0.41) | 30（15-56） | 13.18(6.33-24.53) | 0.95(0.85 to 1.06) | -0.25(-0.40 to -0.10) | -0.12(-0.24 to 0.01) |
| Finland | 50（34-72） | 3.45(2.33-4.98) | 2（2-3） | 0.16(0.12-0.21) | 140（102-187） | 9.81(7.10-13.18) | 28（17-41） | 2.12(1.32-3.16) | 1（0-1） | 0.05(0.03-0.06) | 43（28-65） | 3.36(2.13-5.04) | -1.03(-2.09 to 0.04) | -3.49(-4.35 to -2.63) | -2.91(-3.82 to -1.99) |
| France | 661（445-942） | 4.42(2.97-6.30) | 18（14-24） | 0.13(0.09-0.16) | 1,264（886-1,732） | 8.51(5.96-11.66) | 860（540-1,279） | 5.24(3.27-7.84) | 8（6-11） | 0.05(0.03-0.07) | 826（500-1,262） | 5.04(3.02-7.78) | 0.73(0.31 to 1.15) | -3.17(-3.43 to -2.91) | -1.68(-1.94 to -1.43) |
| Gabon | 1（1-2） | 0.67(0.31-1.26) | 0（0-0） | 0.12(0.06-0.22) | 11（5-20） | 6.30(3.08-11.61) | 4（2-8） | 0.93(0.37-1.96) | 0（0-1） | 0.09(0.04-0.18) | 21（9-43） | 4.85(2.05-10.14) | 0.79(0.63 to 0.96) | -1.21(-1.43 to -1.00) | -1.08(-1.30 to -0.87) |
| Georgia | 29（19-45） | 2.18(1.38-3.31) | 2（1-3） | 0.15(0.11-0.21) | 114（79-164） | 8.54(5.91-12.23) | 41（27-62） | 4.46(2.85-6.72) | 2（2-3） | 0.24(0.17-0.33) | 131（89-183） | 14.19(9.60-19.88) | 2.55(1.34 to 3.77) | 2.06(0.89 to 3.24) | 2.10(0.92 to 3.30) |
| Germany | 969（661-1,384） | 4.67(3.19-6.68) | 28（22-36） | 0.13(0.10-0.17) | 1,869（1,350-2,518） | 8.99(6.48-12.13) | 792（510-1,164） | 3.94(2.52-5.80) | 10（7-14） | 0.05(0.04-0.07) | 892（562-1,341） | 4.44(2.78-6.72) | 0.08(-0.59 to 0.75) | -2.48(-2.91 to -2.05) | -1.57(-2.08 to -1.06) |
| Ghana | 1（1-2） | 0.03(0.02-0.06) | 0（0-0） | 0.01(0.00-0.01) | 9（4-16） | 0.29(0.14-0.53) | 5（2-10） | 0.06(0.02-0.12) | 0（0-1） | 0.01(0.00-0.01) | 27（12-59） | 0.32(0.14-0.68) | 1.88(1.61 to 2.16) | 0.53(0.21 to 0.85) | 0.72(0.38 to 1.06) |
| Greece | 48（35-63） | 1.80(1.33-2.38) | 2（2-2） | 0.07(0.06-0.08) | 119（98-147） | 4.53(3.73-5.58) | 53（39-70） | 1.85(1.36-2.44) | 1（1-2） | 0.04(0.04-0.05) | 89（69-116） | 3.12(2.41-4.09) | 0.05(-0.27 to 0.37) | -1.61(-1.78 to -1.43) | -1.24(-1.42 to -1.05) |
| Greenland | 0（0-1） | 2.66(1.25-4.78) | 0（0-0） | 0.23(0.11-0.38) | 2（1-3） | 13.29(6.41-22.97) | 0（0-1） | 1.99(0.92-4.08) | 0（0-0） | 0.09(0.04-0.18) | 1（0-1） | 5.41(2.60-11.23) | 0.61(-0.25 to 1.48) | -1.76(-2.57 to -0.95) | -1.59(-2.44 to -0.73) |
| Grenada | 0（0-1） | 2.73(1.75-4.12) | 0（0-0） | 0.31(0.21-0.43) | 3（2-4） | 17.23(11.81-24.39) | 1（1-1） | 3.50(2.15-5.40) | 0（0-0） | 0.22(0.15-0.32) | 3（2-5） | 13.01(8.51-19.15) | 1.04(0.51 to 1.58) | -0.46(-1.12 to 0.21) | -0.35(-1.00 to 0.30) |
| Guam | 0（0-0） | 0.48(0.28-1.05) | 0（0-0） | 0.02(0.01-0.05) | 0（0-1） | 1.40(0.88-2.98) | 1（0-1） | 1.43(0.93-2.06) | 0（0-0） | 0.05(0.03-0.06) | 1（1-2） | 2.96(2.03-4.09) | 4.32(3.25 to 5.40) | 2.92(2.28 to 3.57) | 3.17(2.45 to 3.90) |
| Guatemala | 19（15-25） | 1.29(0.99-1.66) | 3（3-4） | 0.23(0.21-0.27) | 186（162-212） | 12.42(10.78-14.20) | 85（61-115） | 2.18(1.56-2.96) | 7（6-9） | 0.19(0.15-0.23) | 399（318-492） | 10.27(8.16-12.68) | 1.23(0.93 to 1.53) | -1.24(-1.68 to -0.80) | -1.06(-1.50 to -0.63) |
| Guinea | 7（3-12） | 0.52(0.25-0.96) | 1（1-2） | 0.10(0.05-0.19) | 75（36-138） | 5.87(2.91-10.70) | 22（9-47） | 0.73(0.31-1.54) | 3（1-6） | 0.10(0.05-0.21) | 182（81-379） | 5.94(2.67-12.16) | 1.09(1.02 to 1.16) | -0.01(-0.06 to 0.04) | 0.10(0.05 to 0.16) |
| Guinea-Bissau | 1（1-2） | 0.52(0.23-1.01) | 0（0-0） | 0.12(0.06-0.23) | 13（6-25） | 6.62(3.07-12.51) | 3（1-5） | 0.56(0.26-1.06) | 0（0-1） | 0.09(0.04-0.16) | 22（10-41） | 4.81(2.24-8.89) | 0.01(-0.07 to 0.10) | -1.28(-1.37 to -1.20) | -1.24(-1.33 to -1.15) |
| Guyana | 1（1-2） | 0.84(0.53-1.26) | 0（0-0） | 0.14(0.10-0.20) | 12（8-18） | 7.51(5.10-10.73) | 3（2-6） | 1.77(0.99-2.94) | 0（0-1） | 0.18(0.11-0.29) | 20（12-31） | 10.13(6.10-16.07) | 2.44(2.19 to 2.69) | 1.14(0.87 to 1.41) | 1.21(0.95 to 1.48) |
| Haiti | 17（7-33） | 1.30(0.57-2.45) | 4（2-8） | 0.33(0.15-0.60) | 229（98-430） | 17.34(7.68-32.19) | 48（21-93） | 1.43(0.65-2.76) | 8（4-15） | 0.24(0.11-0.45) | 434（200-813） | 12.99(6.06-24.21) | 0.53(0.41 to 0.65) | -0.78(-0.87 to -0.68) | -0.71(-0.80 to -0.61) |
| Honduras | 5（2-8） | 0.54(0.29-0.97) | 1（0-1） | 0.09(0.05-0.15) | 40（22-68） | 4.76(2.66-8.01) | 20（9-38） | 0.79(0.37-1.53) | 2（1-4） | 0.08(0.04-0.15) | 105（49-198） | 4.29(2.05-8.02) | 0.87(0.50 to 1.23) | -0.65(-0.91 to -0.40) | -0.67(-0.94 to -0.41) |
| Hungary | 120（79-171） | 4.15(2.74-5.95) | 7（5-9） | 0.24(0.18-0.32) | 403（294-535） | 13.98(10.16-18.66) | 81（54-117） | 2.86(1.87-4.14) | 2（2-3） | 0.08(0.06-0.11) | 151（105-208） | 5.28(3.61-7.40) | -1.72(-2.13 to -1.30) | -3.90(-4.17 to -3.62) | -3.46(-3.75 to -3.16) |
| Iceland | 5（3-7） | 8.06(5.31-11.65) | 0（0-0） | 0.30(0.22-0.40) | 12（8-16） | 18.82(13.51-25.78) | 4（3-6） | 4.76(3.01-7.09) | 0（0-0） | 0.08(0.06-0.11) | 6（4-8） | 6.42(4.19-9.42) | -1.32(-2.36 to -0.28) | -3.87(-4.69 to -3.05) | -3.12(-4.01 to -2.22) |
| India | 2,725（2,002-3,983） | 1.42(1.05-2.07) | 447（324-662） | 0.24(0.17-0.35) | 26,499（19,154-39,198） | 13.68(9.93-20.13) | 11,727（8,484-16,205） | 3.15(2.28-4.34) | 928（676-1,278） | 0.25(0.18-0.34) | 56,440（40,730-78,843） | 15.12(10.92-21.05) | 2.74(2.63 to 2.84) | 0.23(0.12 to 0.34) | 0.36(0.23 to 0.48) |
| Indonesia | 771（453-1,078） | 1.88(1.11-2.62) | 94（55-128） | 0.24(0.14-0.32) | 5,225（3,058-7,213） | 12.79(7.55-17.58) | 2,333（1,391-3,575） | 2.92(1.74-4.48) | 150（89-225） | 0.19(0.11-0.28) | 8,499（5,054-12,935） | 10.60(6.31-16.17) | 1.21(1.05 to 1.37) | -0.85(-0.99 to -0.70) | -0.70(-0.84 to -0.56) |
| Iran | 153（78-254） | 1.52(0.76-2.51) | 5（3-7） | 0.05(0.03-0.07) | 322（183-501） | 3.19(1.80-4.91) | 1,174（445-1,807） | 4.47(1.73-6.87) | 17（7-24） | 0.07(0.03-0.09) | 1,443（583-2,153） | 5.59(2.30-8.33) | 4.51(4.03 to 4.99) | 2.35(1.73 to 2.98) | 3.07(2.49 to 3.66) |
| Iraq | 114（51-216） | 3.43(1.55-6.47) | 6（3-11） | 0.18(0.09-0.33) | 377（174-695） | 11.17(5.25-20.44) | 628（305-1,190） | 6.43(3.14-12.12) | 15（8-26） | 0.15(0.08-0.27) | 1,083（530-1,990） | 11.03(5.44-20.15) | 2.47(2.24 to 2.70) | -0.40(-0.59 to -0.21) | 0.15(-0.03 to 0.33) |
| Ireland | 17（11-24） | 1.95(1.28-2.83) | 1（1-1） | 0.10(0.07-0.13) | 50（36-69） | 5.86(4.21-8.06) | 32（20-48） | 2.23(1.42-3.34) | 1（0-1） | 0.04(0.03-0.06) | 46（30-68） | 3.19(2.07-4.77) | 0.68(0.13 to 1.22) | -2.87(-3.23 to -2.52) | -2.02(-2.40 to -1.63) |
| Israel | 27（18-40） | 2.33(1.54-3.44) | 2（1-2） | 0.14(0.10-0.19) | 93（65-127） | 8.07(5.69-11.05) | 52（34-77） | 2.18(1.42-3.26) | 1（1-2） | 0.05(0.04-0.07) | 89（59-127） | 3.73(2.48-5.36) | 0.39(-0.53 to 1.32) | -2.69(-3.36 to -2.01) | -2.05(-2.78 to -1.31) |
| Italy | 940（711-1,238） | 6.24(4.72-8.22) | 23（21-25） | 0.15(0.14-0.16) | 1,572（1,345-1,867） | 10.44(8.93-12.40) | 832（620-1,076） | 5.06(3.76-6.55) | 9（9-11） | 0.06(0.05-0.06) | 859（676-1,099） | 5.23(4.10-6.71) | -0.84(-1.29 to -0.39) | -3.29(-3.48 to -3.10) | -2.34(-2.58 to -2.09) |
| Jamaica | 7（5-10） | 1.48(0.98-2.18) | 1（0-1） | 0.12(0.09-0.17) | 32（22-44） | 6.85(4.79-9.42) | 25（14-42） | 3.31(1.87-5.45) | 1（1-2） | 0.18(0.11-0.28) | 82（49-129） | 10.73(6.49-16.92) | 2.62(2.12 to 3.13) | 1.35(0.77 to 1.93) | 1.47(0.90 to 2.04) |
| Japan | 1,418（1,092-1,813） | 3.85(2.96-4.92) | 25（24-27） | 0.07(0.06-0.07) | 1,939（1,646-2,337） | 5.23(4.43-6.31) | 1,628（1,253-1,985） | 5.10(3.96-6.15) | 16（16-17） | 0.05(0.05-0.05) | 1,601（1,275-2,033） | 4.99(3.97-6.32) | 0.74(0.46 to 1.01) | -1.23(-1.41 to -1.05) | -0.31(-0.51 to -0.12) |
| Jordan | 31（15-57） | 4.82(2.33-8.76) | 1（1-2） | 0.21(0.11-0.36) | 88（43-154） | 13.25(6.61-22.84) | 161（77-314） | 5.60(2.68-10.89) | 3（2-6） | 0.10(0.05-0.19) | 234（112-452） | 8.09(3.87-15.57) | 0.50(0.12 to 0.89) | -2.60(-2.97 to -2.24) | -1.86(-2.21 to -1.51) |
| Kazakhstan | 157（118-205） | 4.22(3.17-5.53) | 13（12-15） | 0.38(0.33-0.43) | 765（664-884） | 20.80(18.01-24.10) | 202（144-275） | 3.89(2.76-5.30) | 10（8-12） | 0.19(0.15-0.23) | 582（457-730） | 11.24(8.84-14.10) | -0.47(-1.33 to 0.39) | -2.96(-3.63 to -2.30) | -2.64(-3.31 to -1.97) |
| Kenya | 25（15-44） | 0.56(0.33-1.00) | 3（2-6） | 0.08(0.05-0.14) | 210（124-368） | 4.62(2.74-8.26) | 122（69-232） | 1.03(0.59-1.93) | 12（7-23） | 0.10(0.06-0.19) | 729（416-1,398） | 6.07(3.51-11.48) | 2.22(2.05 to 2.38) | 1.37(1.11 to 1.62) | 1.40(1.15 to 1.65) |
| Kiribati | 0（0-0） | 0.02(0.01-0.05) | 0（0-0） | 0.00(0.00-0.01) | 0（0-0） | 0.15(0.06-0.41) | 0（0-0） | 0.02(0.01-0.07) | 0（0-0） | 0.00(0.00-0.01) | 0（0-0） | 0.13(0.05-0.40) | 0.48(0.21 to 0.75) | -0.85(-1.01 to -0.69) | -0.76(-0.93 to -0.60) |
| Kuwait | 27（17-40） | 7.43(4.67-11.02) | 1（0-1） | 0.17(0.12-0.23) | 45（29-65） | 12.36(8.16-17.72) | 108（66-165） | 6.17(3.79-9.47) | 1（1-2） | 0.08(0.05-0.11) | 117（72-182） | 6.87(4.20-10.64) | -1.14(-2.37 to 0.11) | -3.18(-4.40 to -1.96) | -2.49(-3.71 to -1.26) |
| Kyrgyzstan | 36（23-54） | 3.86(2.50-5.78) | 3（2-5） | 0.38(0.26-0.52) | 202（137-286） | 21.72(14.97-30.42) | 56（34-85） | 3.32(2.04-5.06) | 3（2-5） | 0.20(0.13-0.28) | 191（127-276） | 11.44(7.63-16.47) | 0.31(-0.86 to 1.49) | -1.52(-2.52 to -0.51) | -1.40(-2.45 to -0.34) |
| Laos | 14（5-27） | 1.76(0.67-3.40) | 3（1-5） | 0.35(0.14-0.67) | 143（54-278） | 18.13(6.97-35.04) | 56（26-110） | 3.06(1.41-5.94) | 5（2-9） | 0.26(0.12-0.48) | 261（122-483） | 14.32(6.75-26.36) | 1.62(1.54 to 1.70) | -1.26(-1.36 to -1.17) | -1.03(-1.12 to -0.93) |
| Latvia | 25（16-37） | 3.65(2.35-5.41) | 1（1-2） | 0.19(0.13-0.26) | 81（56-113） | 11.66(8.05-16.38) | 14（9-20） | 2.75(1.71-4.11) | 0（0-1） | 0.09(0.07-0.13) | 30（20-44） | 5.96(3.95-8.75) | -1.55(-2.01 to -1.10) | -2.81(-3.29 to -2.32) | -2.73(-3.20 to -2.26) |
| Lebanon | 33（15-62） | 4.78(2.22-8.89) | 1（1-2） | 0.19(0.09-0.33) | 84（40-152） | 11.99(5.75-21.65) | 92（46-168） | 5.73(2.86-10.50) | 1（1-2） | 0.09(0.05-0.16) | 117（57-217） | 7.38(3.62-13.68) | 0.64(0.52 to 0.75) | -2.50(-2.62 to -2.37) | -1.58(-1.71 to -1.46) |
| Lesotho | 2（1-3） | 0.45(0.17-1.03) | 0（0-1） | 0.08(0.03-0.18) | 14（6-32） | 4.21(1.72-9.66) | 5（2-9） | 1.07(0.44-2.15) | 1（0-1） | 0.17(0.08-0.34) | 37（16-75） | 8.86(3.94-17.72) | 4.12(3.42 to 4.82) | 3.85(3.09 to 4.61) | 3.86(3.11 to 4.62) |
| Liberia | 2（1-3） | 0.34(0.14-0.68) | 0（0-1） | 0.07(0.03-0.13) | 17（7-35） | 3.67(1.55-7.27) | 7（3-14） | 0.55(0.24-1.08) | 1（0-1） | 0.06(0.03-0.11) | 42（19-81） | 3.36(1.49-6.39) | 1.47(1.29 to 1.66) | -0.70(-0.95 to -0.45) | -0.54(-0.79 to -0.29) |
| Libya | 48（23-91） | 6.70(3.24-12.60) | 2（1-4） | 0.28(0.15-0.51) | 131（65-246） | 18.13(9.12-33.26) | 247（112-472） | 11.73(5.26-22.48) | 6（3-11） | 0.29(0.14-0.52) | 440（205-813） | 20.93(9.69-38.85) | 2.42(2.05 to 2.79) | 0.43(0.28 to 0.58) | 0.94(0.73 to 1.15) |
| Lithuania | 49（32-71） | 5.11(3.32-7.50) | 2（1-2） | 0.18(0.13-0.24) | 111（78-158） | 11.66(8.13-16.59) | 22（14-32） | 3.02(1.93-4.47) | 1（0-1） | 0.07(0.05-0.10) | 36（24-51） | 5.03(3.36-7.13) | -1.61(-2.33 to -0.89) | -2.66(-3.40 to -1.91) | -2.49(-3.23 to -1.74) |
| Luxembourg | 3（2-4） | 2.70(2.01-3.56) | 0（0-0） | 0.14(0.12-0.16) | 9（7-10） | 8.44(7.07-9.98) | 4（3-5） | 2.04(1.49-2.71) | 0（0-0） | 0.04(0.04-0.05) | 6（4-8） | 3.15(2.36-4.22) | -0.83(-1.25 to -0.40) | -3.87(-4.08 to -3.66) | -3.19(-3.43 to -2.96) |
| Macedonia | 10（7-14） | 1.94(1.27-2.76) | 1（1-1） | 0.14(0.10-0.19) | 41（29-55） | 8.16(5.77-10.75) | 12（7-19） | 1.86(1.13-3.02) | 0（0-1） | 0.07(0.04-0.10) | 27（17-42） | 4.25(2.71-6.71) | -0.11(-0.45 to 0.24) | -2.71(-2.94 to -2.48) | -2.30(-2.53 to -2.07) |
| Madagascar | 44（21-81） | 1.82(0.89-3.31) | 8（4-14） | 0.35(0.18-0.60) | 488（241-860） | 19.87(10.11-34.52) | 162（70-320） | 2.43(1.07-4.80) | 21（10-40） | 0.32(0.15-0.60) | 1,283（588-2,435） | 18.91(8.80-35.59) | 0.88(0.70 to 1.06) | -0.23(-0.40 to -0.06) | -0.16(-0.33 to 0.01) |
| Malawi | 36（17-68） | 1.77(0.85-3.28) | 7（4-13） | 0.35(0.18-0.63) | 421（212-790） | 20.13(10.35-36.95) | 133（55-291） | 2.99(1.27-6.35) | 16（7-35） | 0.38(0.17-0.78) | 1,011（438-2,200） | 22.16(9.91-46.99) | 1.80(1.63 to 1.97) | 0.31(0.08 to 0.53) | 0.38(0.15 to 0.60) |
| Malaysia | 117（57-222） | 2.90(1.45-5.41) | 9（5-16） | 0.24(0.13-0.41) | 551（280-996） | 13.80(7.19-24.42) | 463（229-861） | 5.57(2.77-10.31) | 16（9-28） | 0.19(0.11-0.34) | 1,041（537-1,913） | 12.62(6.56-23.04) | 2.16(1.99 to 2.34) | -0.65(-0.95 to -0.36) | -0.28(-0.58 to 0.03) |
| Maldives | 0（0-1） | 1.20(0.36-2.38) | 0（0-0） | 0.14(0.05-0.28) | 3（1-5） | 7.50(2.33-14.72) | 2（1-3） | 1.38(0.66-2.73) | 0（0-0） | 0.04(0.02-0.08) | 3（2-6） | 2.72(1.34-5.39) | 0.31(-0.04 to 0.65) | -4.31(-4.45 to -4.16) | -3.54(-3.64 to -3.43) |
| Mali | 27（14-49） | 1.59(0.82-2.82) | 5（3-9） | 0.31(0.17-0.53) | 303（160-537） | 17.34(9.24-30.13) | 88（37-182） | 1.87(0.81-3.83) | 11（5-22） | 0.24(0.11-0.48) | 659（284-1,360） | 13.74(6.04-28.08) | 0.48(0.41 to 0.55) | -0.87(-0.97 to -0.77) | -0.77(-0.88 to -0.67) |
| Malta | 3（2-4） | 2.39(1.55-3.51) | 0（0-0） | 0.13(0.09-0.17) | 8（6-11） | 7.58(5.31-10.41) | 4（2-5） | 3.06(1.95-4.66) | 0（0-0） | 0.07(0.05-0.10) | 6（4-8） | 4.91(3.22-7.12) | 0.82(-0.01 to 1.67) | -2.14(-2.76 to -1.51) | -1.49(-2.17 to -0.80) |
| Marshall Islands | 0（0-0） | 0.67(0.33-1.19) | 0（0-0） | 0.09(0.05-0.15) | 0（0-1） | 4.75(2.47-8.28) | 0（0-0） | 1.35(0.59-2.61) | 0（0-0） | 0.11(0.05-0.21) | 1（0-2） | 6.39(2.88-12.20) | 2.13(1.84 to 2.42) | 0.67(0.37 to 0.98) | 0.83(0.52 to 1.13) |
| Mauritania | 1（1-3） | 0.34(0.14-0.66) | 0（0-0） | 0.06(0.03-0.12) | 13（6-26） | 3.35(1.52-6.40) | 5（2-10） | 0.50(0.21-1.04) | 0（0-1） | 0.04(0.02-0.08) | 22（10-45） | 2.39(1.08-4.91) | 0.79(0.58 to 0.99) | -1.64(-1.79 to -1.50) | -1.47(-1.62 to -1.32) |
| Mauritius | 5（3-6） | 1.72(1.27-2.27) | 0（0-0） | 0.12(0.10-0.14) | 18（15-21） | 6.78(5.69-8.11) | 7（5-9） | 1.88(1.36-2.49) | 0（0-0） | 0.07(0.06-0.08) | 15（12-19） | 4.26(3.36-5.31) | 1.16(-0.02 to 2.35) | -0.66(-1.77 to 0.47) | -0.36(-1.48 to 0.78) |
| Mexico | 242（224-260） | 1.41(1.30-1.51) | 28（27-30） | 0.17(0.16-0.18) | 1,554（1,473-1,644） | 9.01(8.54-9.53) | 999（811-1,195） | 2.74(2.23-3.28) | 59（49-70） | 0.16(0.13-0.19) | 3,399（2,775-4,063） | 9.31(7.60-11.12) | 2.29(2.09 to 2.49) | 0.09(-0.27 to 0.45) | 0.35(0.01 to 0.69) |
| Moldova | 15（12-20） | 1.38(1.04-1.82) | 1（1-1） | 0.09(0.08-0.10) | 55（46-66） | 5.01(4.21-6.00) | 17（12-23） | 1.58(1.14-2.14) | 1（1-1） | 0.05(0.05-0.07) | 38（29-49） | 3.49(2.68-4.57) | 0.36(-0.01 to 0.73) | -1.53(-2.02 to -1.04) | -1.21(-1.65 to -0.76) |
| Monaco | 0（0-0） | 2.61(1.29-4.79) | 0（0-0） | 0.10(0.05-0.17) | 1（0-1） | 6.17(3.18-11.00) | 0（0-1） | 3.99(1.82-7.53) | 0（0-0） | 0.08(0.04-0.14) | 1（0-1） | 6.04(2.79-11.07) | 1.63(1.41 to 1.85) | -0.56(-0.65 to -0.48) | 0.07(-0.04 to 0.18) |
| Mongolia | 5（3-10） | 1.45(0.73-2.56) | 1（0-2） | 0.24(0.13-0.40) | 48（24-84） | 12.63(6.52-21.67) | 19（10-34） | 2.12(1.13-3.68) | 2（1-3） | 0.17(0.10-0.27) | 85（48-140） | 9.34(5.29-15.45) | 1.15(0.51 to 1.78) | -1.75(-2.34 to -1.16) | -1.51(-2.11 to -0.91) |
| Montenegro | 6（4-9） | 3.66(2.37-5.54) | 0（0-0） | 0.14(0.10-0.20) | 13（10-19） | 8.80(6.26-12.73) | 6（4-9） | 3.46(2.22-5.34) | 0（0-0） | 0.09(0.07-0.13) | 11（7-16） | 6.33(4.24-9.70) | 0.39(0.10 to 0.68) | -1.12(-1.52 to -0.71) | -0.78(-1.15 to -0.39) |
| Morocco | 101（49-193） | 1.83(0.91-3.45) | 7（3-12） | 0.12(0.07-0.22) | 419（209-776） | 7.45(3.81-13.58) | 341（156-712） | 3.44(1.57-7.20) | 12（6-23） | 0.12(0.06-0.23) | 757（351-1,525） | 7.64(3.53-15.45) | 1.99(1.85 to 2.14) | -0.20(-0.40 to -0.00) | 0.06(-0.13 to 0.26) |
| Mozambique | 41（17-95） | 1.43(0.61-3.25) | 9（4-19） | 0.30(0.14-0.66) | 500（226-1,125） | 17.11(7.83-37.99) | 155（60-391） | 2.31(0.90-5.78) | 23（9-57） | 0.35(0.14-0.86) | 1,380（540-3,411） | 20.00(7.92-49.50) | 1.73(1.62 to 1.84) | 0.81(0.64 to 0.98) | 0.85(0.69 to 1.01) |
| Myanmar | 196（82-382） | 2.22(0.96-4.27) | 30（14-58） | 0.36(0.17-0.68) | 1,693（736-3,287） | 19.33(8.64-37.02) | 466（224-872） | 3.08(1.48-5.77) | 33（17-58） | 0.22(0.11-0.38) | 1,861（930-3,355） | 12.26(6.12-22.12) | 0.68(0.50 to 0.86) | -2.13(-2.35 to -1.90) | -1.95(-2.16 to -1.73) |
| Namibia | 2（1-4） | 0.78(0.37-1.48) | 0（0-1） | 0.13(0.06-0.23) | 20（10-38） | 6.98(3.46-12.88) | 9（4-19） | 1.52(0.65-3.07) | 1（0-2） | 0.14(0.06-0.28) | 50（21-100） | 8.12(3.55-16.20) | 2.25(2.09 to 2.41) | 0.36(0.12 to 0.60) | 0.50(0.28 to 0.73) |
| Nauru | 0（0-0） | 1.12(0.45-2.20) | 0（0-0） | 0.13(0.05-0.24) | 0（0-0） | 6.98(2.95-13.33) | 0（0-0） | 2.04(0.73-4.12) | 0（0-0） | 0.14(0.05-0.27) | 0（0-0） | 8.19(3.04-15.96) | 1.82(1.74 to 1.90) | 0.37(0.14 to 0.59) | 0.52(0.32 to 0.73) |
| Nepal | 57（23-114） | 1.35(0.55-2.66) | 11（4-20） | 0.26(0.11-0.49) | 635（260-1,225） | 14.71(6.16-28.13) | 247（103-517） | 2.85(1.20-5.87) | 22（9-44） | 0.25(0.11-0.50) | 1,311（556-2,732） | 14.98(6.48-30.66) | 2.53(2.43 to 2.64) | 0.01(-0.15 to 0.18) | 0.14(-0.01 to 0.29) |
| Netherlands | 75（50-109） | 1.82(1.21-2.63) | 5（3-6） | 0.11(0.08-0.15) | 271（194-368） | 6.59(4.73-8.97) | 87（55-128） | 2.12(1.32-3.14) | 3（2-4） | 0.07(0.05-0.09) | 183（123-262） | 4.44(2.97-6.41) | 0.98(0.58 to 1.38) | -1.49(-1.73 to -1.24) | -1.04(-1.29 to -0.79) |
| New Zealand | 14（10-21） | 1.60(1.07-2.32) | 1（0-1） | 0.07(0.05-0.09) | 36（26-49） | 4.04(2.93-5.48) | 19（12-27） | 1.41(0.94-2.03) | 0（0-1） | 0.03(0.02-0.04) | 30（20-42） | 2.25(1.53-3.16) | 0.24(-1.04 to 1.53) | -2.04(-3.27 to -0.81) | -1.50(-2.77 to -0.22) |
| Nicaragua | 7（4-11） | 1.03(0.58-1.64) | 1（0-1） | 0.13(0.08-0.19) | 47（27-73） | 6.76(4.01-10.50) | 29（15-52） | 1.69(0.89-2.99) | 2（1-3） | 0.10(0.06-0.17) | 101（55-174） | 5.86(3.19-10.06) | 2.02(1.81 to 2.24) | -0.47(-0.63 to -0.30) | -0.22(-0.39 to -0.06) |
| Niger | 4（2-9） | 0.28(0.12-0.59) | 1（0-2） | 0.06(0.03-0.12) | 49（21-102） | 3.33(1.47-6.79) | 11（5-23） | 0.27(0.12-0.56) | 2（1-3） | 0.04(0.02-0.08) | 91（40-191） | 2.17(0.97-4.53) | -0.50(-0.65 to -0.36) | -1.95(-2.18 to -1.71) | -1.87(-2.11 to -1.64) |
| Nigeria | 14（8-26） | 0.09(0.05-0.16) | 2（1-4） | 0.01(0.01-0.03) | 139（81-239） | 0.83(0.49-1.44) | 69（35-133） | 0.14(0.07-0.27) | 7（4-12） | 0.01(0.01-0.03) | 398（221-739） | 0.81(0.45-1.48) | 1.57(1.42 to 1.73) | -0.29(-0.40 to -0.18) | -0.16(-0.28 to -0.05) |
| Niue | 0（0-0） | 0.85(0.40-1.60) | 0（0-0） | 0.07(0.03-0.12) | 0（0-0） | 3.89(1.94-7.24) | 0（0-0） | 2.07(1.04-3.97) | 0（0-0） | 0.09(0.04-0.16) | 0（0-0） | 5.62(2.89-10.65) | 1.85(1.55 to 2.16) | -0.18(-0.44 to 0.08) | 0.18(-0.12 to 0.47) |
| North Korea | 119（55-226） | 2.26(1.04-4.27) | 9（5-17） | 0.18(0.09-0.32) | 548（266-1,014） | 10.26(5.00-18.93) | 274（120-517） | 3.89(1.70-7.36) | 11（5-20） | 0.15(0.07-0.28) | 698（323-1,295） | 9.83(4.53-18.31) | 1.89(1.76 to 2.02) | -0.47(-0.52 to -0.42) | -0.15(-0.22 to -0.09) |
| Northern Mariana Islands | 0（0-0） | 0.88(0.41-1.66) | 0（0-0） | 0.05(0.02-0.08) | 0（0-1） | 2.73(1.35-5.03) | 0（0-0） | 1.27(0.65-2.34) | 0（0-0） | 0.04(0.02-0.07) | 0（0-1） | 2.76(1.41-5.01) | 0.80(0.39 to 1.22) | -0.28(-0.66 to 0.10) | -0.04(-0.38 to 0.31) |
| Norway | 20（15-26） | 1.76(1.33-2.32) | 1（1-1） | 0.10(0.09-0.11) | 63（55-72） | 5.66(4.96-6.46) | 13（10-18） | 0.95(0.69-1.27) | 0（0-0） | 0.03(0.02-0.03) | 24（20-30） | 1.75(1.44-2.16) | -1.29(-2.39 to -0.18) | -3.60(-4.47 to -2.72) | -3.15(-4.09 to -2.21) |
| Oman | 6（3-12） | 2.11(0.99-4.04) | 0（0-0） | 0.09(0.05-0.17) | 17（8-32） | 5.73(2.83-10.79) | 38（18-73） | 3.57(1.71-6.82) | 1（0-1） | 0.06(0.03-0.11) | 51（23-99） | 4.93(2.26-9.45) | 2.06(1.69 to 2.44) | -0.80(-1.15 to -0.44) | 0.03(-0.29 to 0.36) |
| Pakistan | 561（325-939） | 2.59(1.52-4.27) | 95（58-154） | 0.44(0.28-0.70) | 5,662（3,439-9,383） | 25.48(15.70-41.50) | 3,101（1,618-5,426） | 5.26(2.76-9.16) | 334（185-563） | 0.57(0.32-0.96) | 20,614（11,308-34,853） | 34.60(19.05-58.09) | 1.94(1.72 to 2.16) | 0.44(0.10 to 0.78) | 0.58(0.24 to 0.92) |
| Palau | 0（0-0） | 1.10(0.52-2.06) | 0（0-0） | 0.08(0.04-0.14) | 0（0-0） | 4.74(2.40-8.36) | 0（0-0） | 1.72(0.78-3.28) | 0（0-0） | 0.07(0.04-0.13) | 0（0-0） | 4.79(2.24-8.85) | 1.34(1.11 to 1.58) | -0.28(-0.37 to -0.19) | 0.10(-0.00 to 0.20) |
| Palestine | 11（5-22） | 3.37(1.61-6.48) | 1（0-1） | 0.17(0.08-0.30) | 34（16-63） | 9.99(4.92-18.41) | 49（25-88） | 4.32(2.20-7.78) | 1（1-2） | 0.10(0.06-0.18) | 84（43-149） | 7.40(3.83-13.01) | 0.88(0.67 to 1.10) | -1.36(-1.52 to -1.20) | -0.84(-1.02 to -0.66) |
| Panama | 19（13-29） | 3.50(2.27-5.17) | 2（1-2） | 0.29(0.21-0.40) | 96（67-133） | 17.20(12.07-23.65) | 33（20-51） | 3.06(1.84-4.80) | 1（1-2） | 0.13(0.09-0.20) | 88（56-132） | 8.20(5.26-12.33) | -1.02(-2.03 to -0.00) | -2.78(-3.81 to -1.74) | -2.74(-3.81 to -1.65) |
| Papua New Guinea | 5（2-10） | 0.59(0.24-1.25) | 1（0-1） | 0.07(0.03-0.15) | 32（13-67） | 3.98(1.63-8.18) | 19（8-46） | 0.78(0.31-1.87) | 2（1-4） | 0.07(0.03-0.16) | 97（41-229） | 3.90(1.67-9.29) | 0.63(0.46 to 0.81) | -0.24(-0.33 to -0.15) | -0.14(-0.24 to -0.04) |
| Paraguay | 14（8-22） | 1.70(0.95-2.79) | 1（1-2） | 0.18(0.11-0.28) | 79（45-126） | 9.88(5.73-15.75) | 42（21-76） | 2.35(1.16-4.22) | 2（1-4） | 0.14(0.08-0.24) | 147（76-254） | 8.25(4.29-14.15) | 1.07(0.94 to 1.20) | -0.70(-0.80 to -0.59) | -0.51(-0.62 to -0.40) |
| Peru | 75（42-124） | 1.67(0.95-2.72) | 10（6-15） | 0.22(0.13-0.34) | 541（320-841） | 11.92(7.14-18.39) | 363（180-646） | 3.73(1.85-6.62) | 16（9-28） | 0.17(0.09-0.29) | 997（521-1,690） | 10.24(5.36-17.36) | 2.68(2.43 to 2.92) | -1.03(-1.30 to -0.76) | -0.63(-0.90 to -0.35) |
| Philippines | 471（311-674） | 3.60(2.38-5.13) | 42（30-55） | 0.33(0.24-0.44) | 2,419（1,732-3,199） | 18.65(13.24-24.67) | 1,415（923-2,149） | 5.17(3.38-7.82) | 87（63-121） | 0.32(0.23-0.44) | 5,008（3,639-7,174） | 18.35(13.35-26.12) | 0.89(0.74 to 1.05) | -0.12(-0.18 to -0.05) | -0.08(-0.16 to 0.00) |
| Poland | 500（384-641） | 5.01(3.85-6.43) | 25（24-27） | 0.26(0.25-0.28) | 1,515（1,390-1,676） | 15.46(14.20-17.08) | 404（298-536） | 3.67(2.71-4.87) | 10（8-11） | 0.08(0.07-0.10) | 660（537-805） | 6.00(4.88-7.32) | -1.42(-1.80 to -1.03) | -4.04(-4.53 to -3.55) | -3.42(-3.90 to -2.94) |
| Portugal | 78（52-113） | 3.01(1.99-4.35) | 3（2-5） | 0.13(0.10-0.17) | 207（146-286） | 7.99(5.64-11.09) | 107（70-160） | 3.50(2.27-5.24) | 2（1-2） | 0.05(0.04-0.07) | 131（85-195） | 4.31(2.77-6.45) | 0.28(-0.21 to 0.76) | -3.14(-3.34 to -2.95) | -2.18(-2.41 to -1.95) |
| Puerto Rico | 19（13-29） | 2.03(1.33-3.01) | 1（1-2） | 0.12(0.09-0.16) | 68（48-92） | 7.06(4.99-9.66) | 22（14-34） | 2.58(1.59-4.00) | 1（0-1） | 0.07(0.05-0.11) | 42（27-64） | 4.93(3.14-7.49) | 1.23(0.76 to 1.71) | -1.26(-1.75 to -0.77) | -0.82(-1.32 to -0.32) |
| Qatar | 5（2-9） | 6.34(3.09-11.97) | 0（0-0） | 0.22(0.12-0.39) | 10（5-19） | 14.29(7.29-26.17) | 34（15-73） | 5.48(2.47-11.64) | 0（0-1） | 0.07(0.03-0.14) | 38（17-82） | 6.23(2.80-13.43) | -0.16(-1.00 to 0.69) | -3.59(-4.29 to -2.88) | -2.46(-3.23 to -1.69) |
| Romania | 122（81-176） | 2.15(1.42-3.11) | 10（7-13） | 0.17(0.12-0.22) | 537（385-728） | 9.50(6.82-12.90) | 104（66-156） | 1.99(1.25-3.01) | 4（3-5） | 0.07(0.05-0.10) | 229（152-331） | 4.35(2.85-6.36) | -0.55(-0.82 to -0.28) | -3.40(-3.67 to -3.13) | -2.93(-3.19 to -2.67) |
| Russian Federation | 1,048（984-1,124） | 2.79(2.62-3.00) | 42（40-44） | 0.12(0.11-0.12) | 2,622（2,404-2,899） | 7.10(6.52-7.84) | 1,603（1,386-1,828） | 3.78(3.27-4.32) | 35（31-40） | 0.08(0.07-0.09) | 2,523（2,131-3,019） | 5.99(5.06-7.16) | 2.31(1.27 to 3.37) | -0.25(-1.20 to 0.72) | 0.44(-0.56 to 1.44) |
| Rwanda | 38（18-70） | 2.62(1.24-4.80) | 9（4-15） | 0.61(0.30-1.09) | 516（247-920） | 34.81(16.75-61.60) | 89（37-198） | 2.71(1.13-5.93) | 10（5-22） | 0.32(0.14-0.67) | 617（270-1,350） | 18.61(8.26-39.99) | -0.46(-0.84 to -0.08) | -2.96(-3.33 to -2.58) | -2.81(-3.18 to -2.45) |
| Saint Kitts and Nevis | 0（0-0） | 1.34(1.01-1.75) | 0（0-0） | 0.18(0.15-0.21) | 1（1-1） | 9.73(8.25-11.48) | 0（0-0） | 1.23(0.81-1.80) | 0（0-0） | 0.08(0.06-0.11) | 1（1-1） | 4.54(3.24-6.10) | -0.43(-1.20 to 0.34) | -2.73(-3.72 to -1.72) | -2.59(-3.59 to -1.59) |
| Saint Lucia | 1（1-1） | 2.94(2.23-3.84) | 0（0-0） | 0.33(0.28-0.38) | 5（4-6） | 17.80(15.24-20.89) | 2（2-3） | 4.41(3.00-6.16) | 0（0-0） | 0.26(0.20-0.34) | 8（6-10） | 15.42(11.75-20.05) | 1.57(1.01 to 2.14) | -0.32(-1.05 to 0.41) | -0.14(-0.84 to 0.57) |
| Saint Vincent and the Grenadines | 1（0-1） | 2.89(2.15-3.80) | 0（0-0） | 0.31(0.27-0.36) | 4（3-4） | 17.28(14.58-20.35) | 2（1-2） | 5.33(3.80-7.21) | 0（0-0） | 0.38(0.30-0.46) | 6（5-8） | 21.58(17.08-26.92) | 2.01(1.57 to 2.46) | 0.70(0.16 to 1.26) | 0.78(0.25 to 1.32) |
| Samoa | 1（0-2） | 2.70(1.24-5.15) | 0（0-0） | 0.24(0.12-0.43) | 4（2-8） | 13.97(6.71-25.82) | 2（1-5） | 5.50(2.33-11.05) | 0（0-0） | 0.28(0.13-0.53) | 8（3-15） | 17.37(7.70-33.77) | 2.23(2.05 to 2.41) | 0.55(0.48 to 0.62) | 0.77(0.71 to 0.83) |
| San Marino | 0（0-0） | 3.31(1.65-5.76) | 0（0-0） | 0.12(0.06-0.19) | 0（0-1） | 7.39(3.79-12.50) | 0（0-1） | 3.03(1.20-5.84) | 0（0-0） | 0.06(0.03-0.11) | 0（0-1） | 4.51(1.74-8.77) | 0.60(0.08 to 1.12) | -1.25(-1.59 to -0.92) | -0.74(-1.12 to -0.36) |
| Sao Tome and Principe | 0（0-0） | 0.04(0.02-0.08) | 0（0-0） | 0.01(0.00-0.01) | 0（0-0） | 0.32(0.14-0.66) | 0（0-0） | 0.07(0.03-0.17) | 0（0-0） | 0.01(0.00-0.01) | 0（0-0） | 0.36(0.14-0.81) | 1.70(1.25 to 2.14) | -0.29(-0.77 to 0.20) | -0.14(-0.64 to 0.36) |
| Saudi Arabia | 110（48-217） | 4.03(1.76-7.95) | 6（3-12） | 0.23(0.11-0.44) | 395（182-744） | 14.30(6.64-27.03) | 1,517（732-2,866） | 13.70(6.62-25.92) | 29（15-54） | 0.27(0.14-0.50) | 2,268（1,086-4,208） | 20.74(9.98-38.59) | 4.92(4.54 to 5.31) | 1.06(0.75 to 1.37) | 1.89(1.59 to 2.20) |
| Senegal | 6（2-12） | 0.38(0.16-0.81) | 1（0-2） | 0.07(0.03-0.14) | 54（23-115） | 3.71(1.60-7.75) | 15（6-33） | 0.43(0.18-0.97) | 2（1-3） | 0.05(0.02-0.10) | 91（39-197） | 2.68(1.18-5.71) | 0.01(-0.17 to 0.19) | -1.32(-1.48 to -1.17) | -1.25(-1.41 to -1.09) |
| Serbia | 38（20-69） | 1.53(0.82-2.80) | 3（2-6） | 0.13(0.07-0.23) | 182（99-313） | 7.43(4.06-12.83) | 45（23-79） | 1.84(0.95-3.29) | 2（1-3） | 0.07(0.04-0.13) | 111（60-192） | 4.58(2.43-8.02) | 0.60(0.35 to 0.85) | -2.14(-2.41 to -1.87) | -1.79(-2.04 to -1.55) |
| Seychelles | 0（0-0） | 1.06(0.55-1.82) | 0（0-0） | 0.09(0.05-0.14) | 1（0-1） | 4.83(2.69-7.94) | 0（0-1） | 1.46(0.72-2.54) | 0（0-0） | 0.06(0.03-0.09) | 1（0-2） | 3.54(1.80-6.02) | 1.25(1.02 to 1.49) | -1.00(-1.19 to -0.81) | -0.65(-0.82 to -0.48) |
| Sierra Leone | 3（1-5） | 0.29(0.12-0.62) | 0（0-1） | 0.05(0.02-0.11) | 25（10-52） | 2.92(1.17-5.94) | 8（3-17） | 0.43(0.18-0.90) | 1（0-2） | 0.05(0.02-0.11) | 58（25-117） | 2.99(1.29-6.03) | 1.27(1.20 to 1.34) | 0.02(-0.11 to 0.14) | 0.12(0.01 to 0.23) |
| Singapore | 32（21-46） | 3.48(2.29-5.03) | 1（1-1） | 0.10(0.07-0.13) | 62（43-87） | 6.81(4.73-9.47) | 51（33-76） | 2.85(1.82-4.26) | 1（0-1） | 0.03(0.02-0.05) | 55（34-85） | 3.15(1.94-4.85) | -0.20(-1.02 to 0.63) | -3.21(-3.78 to -2.65) | -2.16(-2.81 to -1.51) |
| Slovakia | 41（23-68） | 2.98(1.66-4.93) | 3（2-4） | 0.19(0.12-0.31) | 151（90-242） | 11.02(6.59-17.71) | 45（23-80） | 2.82(1.44-5.08) | 1（1-3） | 0.09(0.05-0.16) | 95（50-162） | 5.99(3.09-10.40) | 0.23(0.06 to 0.40) | -2.07(-2.23 to -1.91) | -1.63(-1.80 to -1.47) |
| Slovenia | 14（9-19） | 2.58(1.73-3.70) | 1（1-1） | 0.16(0.12-0.21) | 48（35-66） | 9.29(6.69-12.64) | 7（4-11） | 1.22(0.74-1.87) | 0（0-0） | 0.04(0.03-0.06) | 14（9-21） | 2.53(1.56-3.78) | -2.14(-2.76 to -1.52) | -4.37(-4.87 to -3.86) | -3.98(-4.50 to -3.46) |
| Solomon Islands | 0（0-1） | 0.47(0.15-1.04) | 0（0-0） | 0.07(0.02-0.14) | 2（1-4） | 3.72(1.14-7.86) | 2（1-3） | 1.05(0.48-2.11) | 0（0-0） | 0.09(0.05-0.18) | 8（4-17） | 5.31(2.54-10.56) | 2.47(2.26 to 2.69) | 1.03(0.90 to 1.17) | 1.16(1.02 to 1.30) |
| Somalia | 21（9-42） | 1.38(0.58-2.70) | 5（2-9） | 0.33(0.14-0.62) | 279（119-532） | 18.07(7.86-34.18) | 68（27-139） | 1.59(0.63-3.26) | 13（5-26） | 0.31(0.13-0.62) | 765（314-1,514） | 17.60(7.25-34.69) | 0.48(0.41 to 0.56) | -0.10(-0.16 to -0.04) | -0.05(-0.11 to 0.02) |
| South Africa | 110（83-159） | 1.35(1.02-1.92) | 13（10-18） | 0.16(0.12-0.22) | 720（547-1,040） | 8.78(6.68-12.44) | 208（154-324） | 1.34(0.99-2.07) | 17（13-25） | 0.11(0.08-0.16) | 942（711-1,443） | 6.09(4.60-9.27) | 0.49(-0.17 to 1.14) | -0.63(-1.16 to -0.10) | -0.70(-1.27 to -0.12) |
| South Korea | 345（185-649） | 3.01(1.63-5.65) | 14（8-26） | 0.13(0.07-0.23) | 900（492-1,649） | 7.88(4.36-14.36) | 1,004（525-1,749） | 6.99(3.61-12.29) | 11（6-18） | 0.07(0.04-0.12) | 1,033（540-1,820） | 7.17(3.67-12.79) | 3.88(2.58 to 5.19) | -0.89(-1.72 to -0.04) | 0.74(-0.21 to 1.71) |
| South Sudan | 15（6-32） | 1.32(0.55-2.77) | 3（1-6） | 0.25(0.11-0.50) | 161（67-337） | 14.15(6.15-28.69) | 48（20-104） | 2.27(0.92-4.91) | 6（3-13） | 0.31(0.14-0.64) | 383（168-800） | 17.76(7.74-37.22) | 1.80(1.35 to 2.26) | 0.65(0.20 to 1.09) | 0.75(0.30 to 1.20) |
| Spain | 210（140-307） | 2.20(1.46-3.21) | 11（8-14） | 0.11(0.08-0.15) | 645（461-879） | 6.73(4.81-9.17) | 208（134-310） | 1.60(1.03-2.41) | 5（3-6） | 0.03(0.02-0.05) | 329（214-480） | 2.52(1.61-3.75) | -0.91(-1.41 to -0.41) | -3.68(-3.95 to -3.41) | -3.09(-3.38 to -2.80) |
| Sri Lanka | 98（50-171） | 2.31(1.19-4.04) | 8（4-13） | 0.19(0.10-0.31) | 438（236-746） | 10.49(5.73-17.77) | 240（104-468） | 4.03(1.75-7.89) | 7（3-13） | 0.11(0.05-0.21) | 459（203-875） | 7.69(3.39-14.73) | 1.99(1.75 to 2.22) | -1.60(-1.77 to -1.44) | -0.93(-1.11 to -0.75) |
| Sudan | 57（14-166） | 1.48(0.37-4.17) | 5（1-14） | 0.13(0.03-0.36) | 287（74-832） | 7.30(1.92-20.71) | 304（113-619） | 3.05(1.15-6.16) | 12（5-23） | 0.13(0.05-0.24) | 794（300-1,551） | 7.96(3.06-15.42) | 2.54(2.39 to 2.68) | 0.18(0.05 to 0.31) | 0.51(0.38 to 0.64) |
| Suriname | 1（1-2） | 1.40(0.78-2.31) | 0（0-0） | 0.18(0.11-0.29) | 9（5-14） | 10.06(5.84-15.98) | 3（2-5） | 1.94(0.98-3.49) | 0（0-0） | 0.16(0.09-0.27) | 14（7-24） | 9.26(4.87-15.83) | 1.23(0.94 to 1.51) | -0.29(-0.57 to -0.01) | -0.16(-0.47 to 0.14) |
| Swaziland | 1（1-3） | 0.84(0.36-1.65) | 0（0-0） | 0.14(0.06-0.26) | 11（5-22） | 7.34(3.32-14.01) | 4（1-8） | 1.38(0.51-3.02) | 0（0-1） | 0.17(0.07-0.35) | 24（9-50） | 9.15(3.53-18.86) | 1.96(1.39 to 2.53) | 1.06(0.34 to 1.78) | 1.11(0.40 to 1.82) |
| Sweden | 31（22-42） | 1.30(0.93-1.77) | 2（1-2） | 0.07(0.05-0.09) | 96（72-128） | 4.03(3.02-5.36) | 27（18-39） | 1.09(0.73-1.56) | 1（1-1） | 0.03(0.02-0.04) | 51（35-72） | 2.03(1.37-2.89) | 0.82(-0.13 to 1.79) | -1.46(-2.22 to -0.69) | -0.86(-1.71 to -0.01) |
| Switzerland | 49（32-70） | 2.51(1.65-3.61) | 3（2-4） | 0.15(0.11-0.19) | 166（120-225） | 8.70(6.27-11.77) | 27（17-40） | 1.12(0.71-1.66) | 1（1-1） | 0.03(0.02-0.04) | 47（30-70） | 1.97(1.26-2.96) | -2.18(-3.37 to -0.97) | -5.06(-5.94 to -4.17) | -4.60(-5.55 to -3.65) |
| Syria | 35（12-98） | 1.63(0.54-4.49) | 2（1-5） | 0.09(0.03-0.23) | 115（40-311） | 5.21(1.84-14.01) | 146（60-294） | 3.77(1.52-7.68) | 3（1-6） | 0.08(0.03-0.16) | 236（98-458） | 6.03(2.45-11.88) | 2.91(2.76 to 3.06) | -0.03(-0.24 to 0.19) | 0.63(0.44 to 0.82) |
| Taiwan | 334（220-482） | 6.20(4.11-8.92) | 12（9-15） | 0.23(0.17-0.30) | 785（550-1,076） | 14.95(10.55-20.39) | 658（419-973） | 9.60(6.07-14.32) | 10（7-13） | 0.14(0.10-0.19) | 806（518-1,178） | 11.73(7.45-17.36) | 1.80(1.36 to 2.24) | -1.21(-1.41 to -1.01) | -0.32(-0.55 to -0.08) |
| Tajikistan | 0（0-0） | 0.02(0.01-0.04) | 0（0-0） | 0.00(0.00-0.00) | 1（1-2） | 0.16(0.08-0.28) | 0（0-1） | 0.02(0.01-0.03) | 0（0-0） | 0.00(0.00-0.00) | 2（1-3） | 0.09(0.04-0.15) | -1.72(-2.20 to -1.23) | -2.53(-2.88 to -2.17) | -2.45(-2.83 to -2.07) |
| Tanzania | 95（43-186） | 1.83(0.85-3.52) | 16（8-30） | 0.31(0.15-0.59) | 955（446-1,841） | 17.92(8.58-34.04) | 350（147-725） | 2.59(1.11-5.28) | 39（18-81） | 0.30(0.14-0.60) | 2,389（1,050-5,040） | 17.32(7.77-35.90) | 1.02(0.89 to 1.16) | -0.19(-0.30 to -0.08) | -0.11(-0.22 to 0.00) |
| Thailand | 389（205-668） | 2.72(1.45-4.64) | 27（15-44） | 0.19(0.11-0.32) | 1,573（895-2,658） | 11.12(6.39-18.60) | 1,025（500-1,915） | 5.30(2.54-9.95) | 28（15-49） | 0.14(0.07-0.24) | 1,885（972-3,403） | 9.55(4.84-17.36) | 1.32(0.63 to 2.02) | -1.90(-2.43 to -1.37) | -1.30(-1.87 to -0.73) |
| The Bahamas | 2（1-2） | 2.40(1.78-3.15) | 0（0-0） | 0.22(0.19-0.26) | 8（7-9） | 12.50(10.45-14.82) | 4（3-6） | 3.90(2.59-5.58) | 0（0-0） | 0.23(0.17-0.31) | 16（11-21） | 13.74(10.07-18.48) | 1.97(1.52 to 2.42) | 0.39(-0.24 to 1.03) | 0.55(-0.06 to 1.16) |
| The Gambia | 1（0-2） | 0.46(0.21-0.87) | 0（0-0） | 0.07(0.04-0.13) | 8（4-15） | 4.10(1.97-7.52) | 4（2-9） | 0.78(0.33-1.55) | 0（0-1） | 0.08(0.04-0.16) | 27（12-53） | 4.94(2.20-9.38) | 1.47(1.16 to 1.78) | 0.20(-0.09 to 0.49) | 0.31(0.02 to 0.59) |
| Timor-Leste | 2（1-4） | 1.12(0.51-2.19) | 0（0-1） | 0.18(0.09-0.34) | 15（7-29） | 9.54(4.48-18.27) | 6（3-11） | 2.05(0.98-3.89) | 0（0-1） | 0.17(0.09-0.31) | 26（13-49） | 9.38(4.57-17.51) | 2.07(1.63 to 2.52) | -0.31(-0.77 to 0.17) | -0.12(-0.62 to 0.38) |
| Togo | 3（1-7） | 0.44(0.19-0.89) | 1（0-1） | 0.07(0.03-0.15) | 30（14-60） | 4.08(1.88-8.18) | 10（4-20） | 0.48(0.20-1.01) | 1（0-2） | 0.05(0.02-0.11) | 62（26-124） | 3.07(1.32-6.15) | 0.20(-0.03 to 0.42) | -1.07(-1.19 to -0.95) | -1.00(-1.14 to -0.87) |
| Tokelau | 0（0-0） | 0.85(0.39-1.56) | 0（0-0） | 0.09(0.04-0.16) | 0（0-0） | 4.96(2.43-9.03) | 0（0-0） | 2.22(1.12-4.27) | 0（0-0） | 0.10(0.05-0.18) | 0（0-0） | 6.46(3.28-12.23) | 2.47(2.19 to 2.74) | -0.13(-0.35 to 0.09) | 0.24(-0.02 to 0.51) |
| Tonga | 0（0-0） | 0.66(0.32-1.23) | 0（0-0） | 0.05(0.03-0.09) | 1（0-1） | 2.98(1.52-5.34) | 0（0-1） | 1.06(0.48-2.12) | 0（0-0） | 0.05(0.02-0.09) | 1（0-1） | 3.11(1.45-6.09) | 1.18(0.96 to 1.39) | -0.31(-0.46 to -0.16) | -0.08(-0.23 to 0.07) |
| Trinidad and Tobago | 5（4-6） | 1.74(1.31-2.27) | 1（0-1） | 0.19(0.16-0.22) | 28（24-33） | 10.26(8.79-11.97) | 12（8-18） | 3.17(2.06-4.71) | 1（1-1） | 0.19(0.14-0.26) | 42（30-58） | 11.06(7.78-15.29) | 2.13(1.76 to 2.49) | -0.04(-0.43 to 0.35) | 0.19(-0.20 to 0.57) |
| Tunisia | 62（30-115） | 3.46(1.68-6.40) | 2（1-4） | 0.14(0.07-0.24) | 159（78-292） | 8.87(4.43-15.98) | 217（102-414） | 6.41(3.00-12.34) | 4（2-7） | 0.12(0.06-0.21) | 312（150-583） | 9.30(4.43-17.53) | 1.99(1.82 to 2.17) | -0.49(-0.62 to -0.36) | 0.16(0.02 to 0.29) |
| Turkey | 442（201-811） | 3.58(1.65-6.52) | 24（12-43） | 0.20(0.10-0.35) | 1,503（710-2,662） | 12.10(5.82-21.24) | 1,068（510-1,940） | 4.53(2.15-8.29) | 20（10-34） | 0.08(0.04-0.14) | 1,527（761-2,809） | 6.49(3.20-12.03) | 0.94(0.77 to 1.11) | -2.94(-3.07 to -2.81) | -2.02(-2.15 to -1.90) |
| Turkmenistan | 13（10-16） | 1.81(1.38-2.34) | 1（1-1） | 0.20(0.17-0.22) | 77（67-87） | 10.94(9.57-12.46) | 27（18-39） | 2.15(1.44-3.15) | 2（1-3） | 0.15(0.11-0.21) | 109（79-148） | 8.77(6.37-11.95) | 0.59(-0.35 to 1.54) | -0.93(-1.80 to -0.05) | -0.77(-1.65 to 0.12) |
| Tuvalu | 0（0-0） | 0.72(0.35-1.32) | 0（0-0） | 0.10(0.05-0.18) | 0（0-0） | 5.42(2.73-9.91) | 0（0-0） | 1.36(0.65-2.62) | 0（0-0） | 0.09(0.05-0.16) | 0（0-0） | 5.21(2.66-9.50) | 1.85(1.64 to 2.06) | -0.40(-0.46 to -0.33) | -0.17(-0.24 to -0.10) |
| Uganda | 47（21-89） | 1.41(0.66-2.67) | 8（4-15） | 0.25(0.12-0.45) | 485（233-889） | 14.26(6.98-25.82) | 376（166-758） | 4.01(1.82-7.93) | 42（20-81） | 0.46(0.22-0.86) | 2,621（1,207-5,220） | 27.21(12.88-52.73) | 2.91(2.59 to 3.22) | 1.38(0.96 to 1.81) | 1.50(1.07 to 1.93) |
| Ukraine | 316（202-473） | 2.38(1.52-3.56) | 12（9-17） | 0.09(0.06-0.13) | 753（508-1,078） | 5.66(3.82-8.12) | 400（197-709） | 3.21(1.58-5.70) | 12（6-19） | 0.09(0.05-0.15) | 771（396-1,351） | 6.11(3.12-10.74) | 0.83(0.28 to 1.39) | -0.30(-0.81 to 0.21) | -0.05(-0.57 to 0.47) |
| United Arab Emirates | 15（6-29） | 5.48(2.22-10.55) | 1（0-1） | 0.29(0.12-0.52) | 46（19-87） | 17.03(7.15-31.66) | 125（61-236） | 6.15(2.93-11.60) | 3（2-5） | 0.14(0.07-0.26) | 211（104-386） | 10.60(5.07-19.41) | 0.33(0.01 to 0.65) | -2.14(-2.34 to -1.94) | -1.45(-1.70 to -1.21) |
| United Kingdom | 226（207-248） | 1.52(1.39-1.67) | 12（11-13） | 0.08(0.08-0.08) | 714（665-775） | 4.82(4.49-5.23) | 333（302-368） | 1.90(1.72-2.10) | 11（10-11） | 0.06(0.06-0.06) | 676（612-751） | 3.85(3.49-4.28) | 1.37(1.14 to 1.61) | -0.55(-0.71 to -0.39) | -0.20(-0.37 to -0.02) |
| United States | 2,178（2,027-2,345） | 3.13(2.91-3.37) | 43（41-44） | 0.06(0.06-0.06) | 3,222（2,838-3,705） | 4.66(4.11-5.35) | 3,939（3,617-4,289） | 4.75(4.36-5.17) | 53（51-56） | 0.06(0.06-0.07) | 4,600（3,933-5,471） | 5.57(4.76-6.63) | 1.60(1.40 to 1.80) | 0.33(0.18 to 0.48) | 0.83(0.68 to 0.98) |
| Uruguay | 19（12-29） | 2.50(1.63-3.78) | 1（1-2） | 0.18(0.13-0.25) | 79（55-112） | 10.42(7.22-14.80) | 30（19-45） | 3.36(2.13-5.04) | 1（1-2） | 0.13(0.09-0.18) | 76（51-106） | 8.35(5.66-11.81) | 1.18(0.78 to 1.59) | -0.79(-1.15 to -0.43) | -0.48(-0.86 to -0.11) |
| Uzbekistan | 15（9-22） | 0.39(0.25-0.59) | 1（1-2） | 0.04(0.03-0.05) | 80（55-113） | 2.12(1.48-2.95) | 82（51-124） | 0.89(0.56-1.35) | 6（4-8） | 0.06(0.04-0.09) | 327（221-472） | 3.58(2.42-5.15) | 2.77(2.02 to 3.52) | 1.44(0.77 to 2.10) | 1.55(0.89 to 2.21) |
| Vanuatu | 0（0-0） | 0.54(0.23-1.09) | 0（0-0） | 0.07(0.03-0.13) | 1（0-2） | 3.63(1.60-7.18) | 1（0-1） | 0.94(0.46-1.77) | 0（0-0） | 0.08(0.04-0.14) | 3（2-6） | 4.61(2.41-8.51) | 1.29(1.11 to 1.48) | 0.29(0.14 to 0.43) | 0.38(0.23 to 0.53) |
| Venezuela | 45（34-59） | 1.12(0.86-1.47) | 5（4-5） | 0.12(0.11-0.14) | 265（228-305） | 6.69(5.76-7.73) | 190（122-281） | 2.50(1.61-3.69) | 11（8-15） | 0.15(0.10-0.20) | 644（450-887） | 8.50(5.94-11.71) | 2.59(2.31 to 2.88) | 0.39(0.06 to 0.72) | 0.69(0.35 to 1.02) |
| Vietnam | 434（199-846） | 2.94(1.39-5.60) | 36（17-67） | 0.26(0.13-0.47) | 2,164（1,020-4,086） | 14.86(7.28-27.22) | 2,734（1,226-5,279） | 9.83(4.36-19.09) | 88（43-161） | 0.31(0.15-0.58) | 5,818（2,712-10,880） | 21.02(9.67-39.57) | 4.49(4.26 to 4.72) | 1.20(0.91 to 1.49) | 1.67(1.37 to 1.97) |
| Virgin Islands, U.S. | 0（0-1） | 1.10(0.57-1.96) | 0（0-0） | 0.09(0.05-0.14) | 2（1-3） | 4.98(2.69-8.30) | 0（0-0） | 1.09(0.46-2.32) | 0（0-0） | 0.05(0.02-0.11) | 1（0-1） | 3.16(1.36-6.78) | 0.38(0.15 to 0.61) | -0.98(-1.22 to -0.74) | -0.81(-1.03 to -0.59) |
| Yemen | 19（6-46） | 0.91(0.31-2.25) | 2（1-4） | 0.08(0.03-0.20) | 91（33-219） | 4.39(1.61-10.62) | 148（66-286） | 2.10(0.95-4.00) | 8（4-14） | 0.11(0.05-0.20) | 459（210-842） | 6.55(3.02-11.89) | 3.20(2.83 to 3.57) | 1.29(1.02 to 1.55) | 1.56(1.27 to 1.86) |
| Zambia | 33（16-60） | 2.11(1.03-3.77) | 6（3-11） | 0.42(0.21-0.73) | 390（193-687） | 23.97(12.08-41.76) | 161（51-492） | 3.63(1.20-10.76) | 19（6-57） | 0.43(0.16-1.28) | 1,145（381-3,610） | 25.37(8.83-77.16) | 1.78(1.32 to 2.25) | 0.02(-0.25 to 0.30) | 0.12(-0.16 to 0.40) |
| Zimbabwe | 41（20-76） | 2.15(1.09-3.97) | 5（3-10） | 0.30(0.16-0.53) | 318（161-579） | 16.64(8.73-29.50) | 161（69-326） | 4.46(1.95-8.97) | 22（10-42） | 0.62(0.28-1.18) | 1,250（541-2,453） | 34.41(15.25-66.68) | 3.34(2.26 to 4.43) | 3.87(2.75 to 5.01) | 3.89(2.76 to 5.03) |

ASIR, age-standardized incidence rate; ASMR, age-standardized mortality rate; ASDR, age-standardized DALY rate; DALYs, disability-adjusted life-years; EAPC, estimated annual percentage change; UI, uncertainty interval; CI, confidence interval.
